# Holocene vegetation history and human impact in the eastern Italian Alps: a multi-proxy study on the Coltrondo peat bog, Comelico Superiore, Italy

**DOI:** 10.1007/s00334-019-00749-y

**Published:** 2019-10-01

**Authors:** Michela Segnana, Klaus Oeggl, Luisa Poto, Jacopo Gabrieli, Daniela Festi, Werner Kofler, Piergiorgio Cesco Frare, Claudio Zaccone, Carlo Barbante

**Affiliations:** 1 Department of Environmental Sciences, Informatics and Statistics, Ca’ Foscari University of Venice, via Torino 155, 30172 Venice-Mestre, Italy; 2 Institute for Botany, University of Innsbruck, Sternwarterstraße 15, 6020 Innsbruck, Austria; 3 Institute for the Dynamics of Environmental Processes – CNR, Ca’ Foscari University of Venice, via Torino 155, 30172 Venice-Mestre, Italy; 4 Belluno, Italy; 5 Department of Sciences of Agriculture, Food and Environment, University of Foggia, via Napoli 25, 71121 Foggia, Italy

**Keywords:** Palaeoclimatology, Dolomites, Pollen analysis, Vegetation dynamics, Lead, Mining

## Abstract

The present study aims to reconstruct vegetation development, climate changes and human impact using an ombrotrophic peat core from the Coltrondo bog in the eastern Italian Alps. Evidence from pollen, micro-charcoal, major and trace elements, and lead isotopes from this 7,900 years old peat deposit has been combined, and several climatic oscillations and phases of human impact detected. In particular, human presence was recorded in this area of the Alps from about 650 cal bc, with periods of increased activity at the end of the Middle Ages and also at the end of the 19th century, as evidenced by both human-related pollen and the increase in micro-charcoal particles. The enrichment factor of lead (EF_Pb_) increased since the Roman period and the Middle Ages, suggesting mainly mining activities, whereas the advent of industrialization in the 20th century is marked by the highest EF_Pb_ values in the whole core. The EF_Pb_ data are strongly supported by the ^206^Pb/^207^Pb values and these are in general agreement with the historical information available. Therefore, the multi-proxy approach used here has allowed detection of climatic events and human impact patterns in the Comelico area starting from the Iron Age, giving new insights into the palaeoecology as well as the course of the interaction among humans, climate and ecosystems in this part of the eastern Italian Alps.

**Electronic supplementary material:**

The online version of this article (10.1007/s00334-019-00749-y) contains supplementary material, which is available to authorized users.

## Introduction

The European Alps represent a region, which is highly sensitive to both natural and human influences. It is therefore of crucial importance to reconstruct past environmental conditions and to try to understand how climate and human societies jointly affected (and still affect) this mountain environment. Numerous attempts have been carried out to reconstruct the vegetation and climatic evolution of the western and central Alps using different kinds of records, including those from lakes, peatlands, stalagmites and tree rings (for example, Wick and Tinner 1997; Pini 2002; Tinner and Theurillat 2003; Mangini et al. 2005; Nicolussi et al. 2005; Büntgen et al. 2011; Magny 2013). However, only a few reports are available for the Italian part of the eastern Alps (Fairchild et al. 2001; Frisia et al. 2003; Vescovi et al. 2007; Scholz et al. 2012; Poto et al. 2013), due to the scarcity of well-preserved deposits at high altitudes with high resolution records and adequate chronologies. In addition, many palynological studies date back to the 1980s–1990s (for example, Seiwald 1980; Kral and Carmignola 1986; Kral 1982, 1986a, 1991; Wahlmüller 1990), with only few recent exceptions (Burga and Egloff 2001; Burga and Perret 2013; Poto 2013; Festi et al. 2014). Moreover, our knowledge about early human impact in the investigated area at Comelico is rather poor. The only archaeological remains discovered date back to the Mesolithic (Cesco Frare and Mondini 2005; Visentin et al. 2015) and the absence of later archaeological finds makes the reconstruction of human settlement history in this valley difficult. Historical documentation is also very scarce until the beginning of rule by the Republic of Venice in the 15th century ad.

Peat deposits play an important role in the reconstruction of Holocene climatic and vegetation variations (Charman 2002; Chambers et al. 2012). In particular, ombrotrophic mires, as they receive water and nutrients only from wet and dry atmospheric depositions, provide a valuable record for the study of past environmental changes (Damman 1978; Clymo 1983), whether these are natural or human induced.

In the present study, a peat bog in the Comelico area, in the eastern Italian Alps, was investigated. Data from pollen, non-pollen palynomorphs (NPPs) and micro-charcoal analyses, coupled with physical and geochemical results, were used as proxies to reconstruct past vegetation dynamics, land use changes and human impact in this area, as well as their implications for past climatic variations. This multi-proxy study aims to contribute towards filling an important information gap about this area of the Italian Alps, especially considering the lack of archaeological and historical data there, providing an in-depth understanding of Holocene vegetation dynamics and human impact, including farming and mining activities.

## Study site

**Fig. 1:**
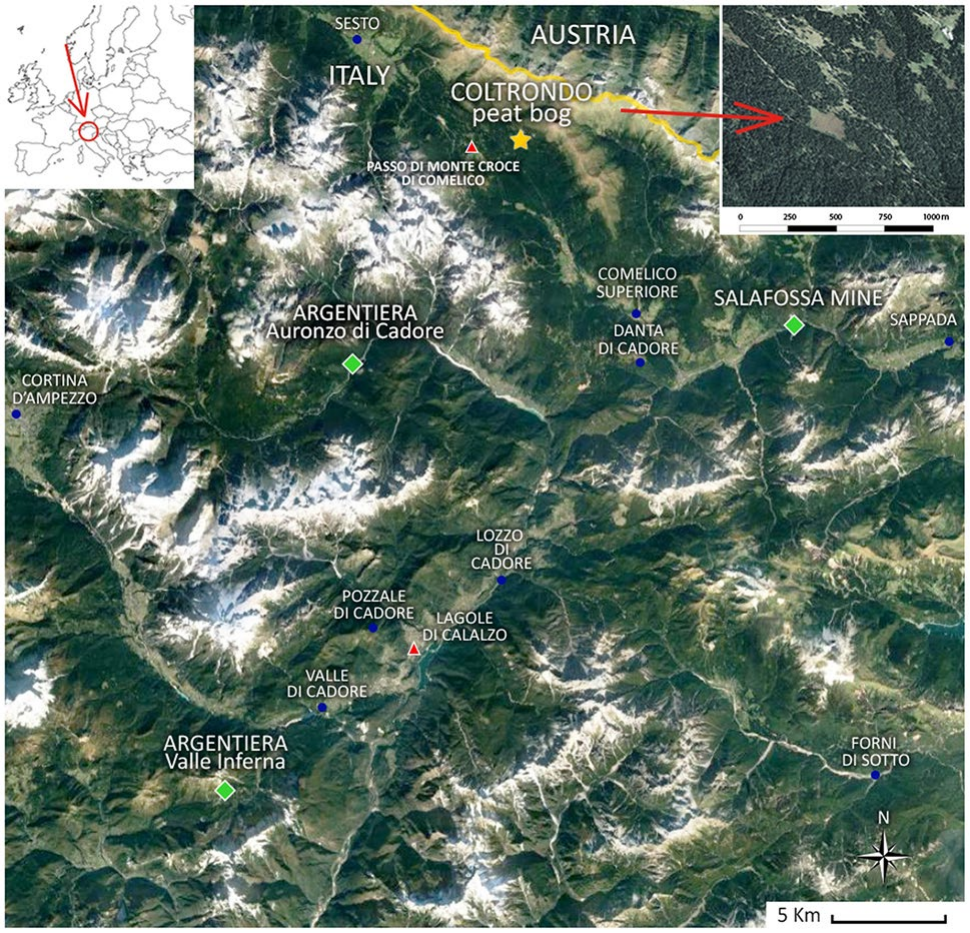
Satellite view of the Cadore area. Yellow star, the Coltrondo peat bog; green diamonds, the main mining sites, Salafossa mine, Argentiera of Auronzo di Cadore, Argentiera of Valle Inferna; blue circles, villages; red triangles, archaeological sites (from CNES/Airbus, Google, DigitalGlobe, European Space Imaging, Dati cartografici, Google)

The Coltrondo peat bog (46°39′28.37″N, 12°26′59.17″E) is located in the Dolomites (Provincia di Belluno, Veneto, Italy), at about 1,790 m a.s.l., in the upper basin of the river Piave on the southwest facing side of the Val Padola in the Comelico area (Fig. 1). The bog covers an area of 3.7 ha and is part of a peatland system, which covers 14 ha, and is one of the most valuable natural sites in the region. The area is included in the European Nature 2000 Network under Special Protection Area “Dolomiti del Cadore e del Comelico” (code number: IT3230089). Detailed information about the geological and geomorphological features of the area is in the ESM.

## Modern vegetation

The vegetation of the Comelico area is characterized by conifer woods dominated by *Picea abies*, from valley floors up to the tree line at about 2,000 m. *Abies alba* is also present but in much smaller amounts, as well as *Larix decidua*, which mainly grows near mountain meadows and in abandoned pastures, while *Pinus sylvestris* is present where soils are poor in nutrients. *Pinus mugo* grows quite extensively in pure stands on steep slopes above the tree line. Modern vegetation has been influenced by centuries of forestry management, which favoured *Picea* over *Abies*, *Pinus*, *Larix* and especially *Fagus sylvatica*, which is present nowadays only as an understory shrub. The abandonment of pastures used for grazing in the last few decades has allowed the spread of shrubs and small trees, such as *Rhododendron*, *Vaccinium*, *Salix* and *Alnus viridis*. The Coltrondo peat bog has a high biodiversity, and is dominated by *Sphagnum* mosses, *Eriophorum vaginatum*, *Calluna vulgaris* and *Vaccinium microcarpum*, with the presence of some rare and endangered plants, including *Drosera intermedia* and *Carex chordorrhiza*. A spruce wood surrounds the peatland, with *Pinus mugo* growing on the margin of the bog (Andrich et al. 2001).

### Palynological studies

Early studies by Kral (1986a) were carried out on Lago di Sant’Anna and the Danta di Cadore peat bog; the latter area was then reinvestigated by Poto (2013), who analyzed the transition from the Last Glacial Maximum to the Holocene, an interval of time not covered by the Coltrondo peat bog. The Holocene vegetation development of the area around the study site has also been covered by some other publications (such as Seiwald 1980; Kral 1982, 1991; Kral and Carmignola 1986; Stumböck 2000; Heiss et al. 2005; Borgatti et al. 2007; Schneider et al. 2010; Festi et al. 2014) (ESM Fig. S1, Table S1). The data on the eastern Italian Alps provided by these works reflect the general climatic trends recorded there, with some major oscillations during the Holocene. Since the first permanent human settlements, the effects of climate change have been masked by human activities, mainly farming and grazing, but also mining, making it difficult to distinguish climatic events from disturbances caused by humans. Moreover, most of these pollen studies date back to the 1980s and lack precise dating, with no ^210^Pb dates and with few, if any, radiocarbon dates, characterized by high statistical uncertainty.

### Archaeological and historical context

The earliest archaeological evidence found in the Comelico area, flint artefacts, dates back to the Mesolithic (Cesco Frare and Mondini 2005; Visentin et al. 2015; Fontana and Visentin 2016), a time when nomadic foragers hunted large herbivores at high altitudes. There are no later archaeological finds, making it difficult to reconstruct with any precision the prehistory of the area and the human settlements in the Val Padola. The evidence of several burial places in the nearby Cadore area (Lozzo, Pozzale and Valle; Fig. 1) indicates that it was already inhabited by the 7th century bc (Ciani 1862) and this is confirmed by the inscriptions from the ancient Veneti, who lived in the area at this time, found at the archaeological site of Lagole di Calalzo (Fig. 1), dating to the 7th century bc (Lomas 2003).

Roman finds are abundant in Cadore, which became subject to Roman rule in 184 bc. Settlements near Sesto and San Candido (Dal Ri and Di Stefano 2005) suggest the presence of a Roman road connecting Auronzo to the Val Pusteria/Pustertal across the Passo di Monte Croce/Kreuzbergpass (De Bon 1938; Fig. 1). Recent finds on the latter (Pirazzini et al. 2014) validate this assumption, as well as the presence of a sacred place on Monte Calvario near Auronzo, in use also as an important Roman territorial garrison since the 2nd century bc (Conte 2013).

Records from the early Middle Ages are extremely scarce for the Cadore area. The earliest available written sources date to the 12th century and refer to the regulation of the extensive pasture in use at that time (Ciani 1862; Collodo 1988). Indeed, the use of pasture and woods were regulated by *laudi* or rural codes which were democratically approved (Rosolo 1982). The *laudi* provided detailed instructions for the use of summer pastures in mountain environments (“montes”), also indicating the alpine routes (“strade delle peccore”) to follow (Cesco Frare 2011). This evidence corresponds most probably to the first colonization of the Comelico area (Cesco Frare 2016).

From ad 1420, the Cadore and Comelico areas were governed by the Republic of Venice, which encouraged mining, which was already being done in the area (Cucagna 1961; Vergani 2003), and the use of woodlands for commercial purposes. This resulted in a general depletion of woodland. At the end of the 18th century, in 1797 the Cadore and Comelico areas fell under the domination of Napoleon, then from 1814 under Austrian control, which lasted until 1866, when the Cadore became part of the Kingdom of Italy (Fabbiani 1992). In the first half of the 20th century, the two World Wars had a significant impact on this area, in terms of damage and depopulation. In the last few decades, the Comelico area has seen a decrease in population and a general abandonment of traditional, centuries-old practices such as farming and grazing.

### Mining activities through the centuries

Although mineral deposits in the eastern Italian Alps have been exploited since prehistoric times, possibly since the Late Neolithic (Artioli et al. 2014, 2015), metallurgical activities in the studied area are scarcely documented prior to the Venetian period. Cucagna (1961) and De Lorenzo (1999) both argue that Roman exploitation of the local mineral resources is more than plausible, due to the Roman presence in the area and the archaeological discovery of possibly local metal artefacts dated ca. 300 bc. De Lorenzo (1999) mentions a decline of this activity after the fall of the Roman Empire and the subsequent invasions, with a revival from the 11th century ad, when historical documents indicate the extraction of the mineral resources in the entire Cadore area and the presence of furnaces for metal processing, mainly iron (Fe), but the importance of silver (Ag) in the medieval economy is not to be overlooked. Therefore, every small Pb–Ag mining site was probably exploited (Vergani 2003). Between ad 1460 and 1530 mining and metal processing activities increased significantly all over Europe, primarily for copper (Cu) and Ag, but also for lead (Pb) and gold (Au). This surge was followed by a decline, partly due to the depletion of the deposits, but mostly because of the import of metals from Central and South America (Vergani 2003). This decline also affected the Cadore area and the nearby province of Vicenza, where Ag extraction had flourished until then. In the following centuries, mining in Cadore had periods of high activity alternating with decline and abandonment, until its irreversible decline from the end of the 18th century, in conjunction with the end of the Republic of Venice and a general economic crisis. In the 19th and 20th centuries, mining continued in Cadore mainly for Pb and zinc (Zn), with various mining concessions alternating with periods of inactivity, but all the mines in the area are abandoned now.

## Materials and methods

### Sampling

In June 2011, a 250 cm peat core was collected from the Coltrondo bog. The top 100 cm was sampled as a monolith with a 15 × 15 × 100 cm modified Wardenaar corer (Wardenaar 1987), whereas the deepest layers were collected using a Belarus corer providing semi-cylindrical peat sections 50 cm long and 10 cm wide (Jowsey 1966), using a two borehole technique (Givelet et al. 2004). The entire core was wrapped in plastic film, brought to the laboratory and stored at − 18 °C immediately after collection. It was subsequently cut while still frozen into 1 cm slices using a stainless steel band saw. Each slice was then divided into several sub-samples for the various parts of the multi-proxy study.

### Dating and age-depth model

The chronology of the core was obtained using different radiometric techniques in order to obtain a reliable and detailed age-depth relationship. ^210^Pb and ^137^Cs measurements were carried out by direct gamma assay at the Liverpool University Environmental Radioactivity Laboratory, UK, using Ortec HPGe GWL series well-type coaxial low background intrinsic germanium detectors (Appleby et al. 1986). Fifteen peat samples taken from the top 40 cm were analysed (Table 1) and ^210^Pb dates were then calculated using the Constant Rate of Supply (CRS) model (Appleby and Oldfield 1978); the Constant Initial Concentration (CIC) model gives similar results.

**Table 1:** Fallout radionuclide concentrations (total ^210^Pb, unsupported ^210^Pb, supported ^210^Pb and ^137^Cs) and ^210^Pb chronology of the uppermost layers of the Coltrondo peat core

Average depth (cm)	Total ^210^Pb (Bq kg^−1^)	Unsupported ^210^Pb (Bq kg^−1^)	Supported ^210^Pb (Bq kg^−1^)	^137^Cs (Bq kg^−1^)	Date (ad)
0					2011 ± 0
3.6	570 ± 57	570 ± 57	0 ± 0	369 ± 13	2005 ± 2
4.5	745 ± 91	745 ± 91	0 ± 0	274 ± 19	2003 ± 2
5.5	412 ± 59	412 ± 59	0 ± 0	147 ± 10	2001 ± 2
6.6	416 ± 56	416 ± 56	0 ± 0	254 ± 12	1999 ± 2
7.6	571 ± 42	571 ± 42	0 ± 0	466 ± 11	1997 ± 2
8.5	574 ± 65	574 ± 65	0 ± 0	346 ± 16	1995 ± 2
9.6	426 ± 41	426 ± 41	0 ± 0	248 ± 9	1992 ± 2
13.7	370 ± 38	370 ± 38	0 ± 0	238 ± 8	1981 ± 3
17.8	306 ± 39	306 ± 39	0 ± 0	103 ± 7	1969 ± 4
21.9	256 ± 51	256 ± 51	0 ± 0	83 ± 9	1960 ± 5
26.0	153 ± 33	153 ± 33	0 ± 0	53 ± 6	1950 ± 6
28.1	106 ± 17	99 ± 17	6 ± 2	29 ± 3	1944 ± 7
30.1	62 ± 19	56 ± 19	6 ± 2	48 ± 3	1935 ± 8
33.5	30 ± 5	23 ± 5	6 ± 2	30 ± 1	1910 ± 10
37.5	32 ± 10	26 ± 10	6 ± 2	19 ± 1	1886 ± 12

Accelerator mass spectrometry (AMS) ^14^C dating was done for the rest of the core; eleven samples (six of plant macrofossil remains and five peat bulk samples) were selected from the core, pre-treated to avoid contamination (with acid/alkali for the plant macroremains and acid for the bulk peat samples), dried and submitted to the ^14^CHRONO Centre, Queens University of Belfast, UK (Table 2). The ^14^C dates were calibrated using Clam v. 2.2 (Blaauw 2010) and the IntCal13 calibration curve (Reimer et al. 2013), within the statistical software R v. 3.2.3 (R Core Team 2015). The age-depth relationship was created by combining the dates obtained from the two techniques, using Clam. The final result of the calibration is expressed as the range within which the probability of finding the calibrated age is 95% and the best value is calculated as the weighted mean of all age-model iterations created by Clam for each depth. Unless otherwise mentioned, all dates in this paper are given in calendar years bc/ad within the 95% confidence interval.

**Table 2:** Radiocarbon ages and calibrated values, with the estimated and the best values, and also the material dated

Average depth (cm)	^14^C age (bp)	Estimated 2σ range (cal bp)	Best value (cal bp)	Analysed fraction	Lab code
45.7	469 ± 27	493–535	515	Cone	UBA-25138
62.5	885 ± 27	680–755	716	Wood	UBA-25139
73.0	899 ± 26	785–901	842	Peat	UBA-26181
73.0	942 ± 26	785–901	842	Wood	UBA-26182
100.1	922 ± 25	2,439–2,516	2,477	Wood	UBA-25140
115.5	3,180 ± 29	3,360–3,451	3,406	Peat	UBA-26183
135.5	4,229 ± 31	4,653–4,850	4,773	Peat	UBA-26184
148.5	4,891 ± 45	5,506–5,719	5,630	Peat	UBA-25143
167.0	5,259 ± 39	5,936–6,174	6,041	Wood	UBA-25144
211.5	6,019 ± 36	6,762–6,950	6,860	Peat	UBA-25142
239.0	6,790 ± 43	7,554–7,687	7,635	Wood	UBA-25141

### Physical and chemical analyses

For all analyses, peat samples were selected at a 1 cm interval for the uppermost 100 cm and at a 3 cm interval for the lower part of the core (n = 145).

Bulk density was calculated by dividing the dry weight of each peat sample, after being dried at 105 °C overnight, by its corresponding volume, measured with a gauge. The ash content is expressed as a percentage of the initial dry weight after combustion in a muffle furnace at 550 °C for 5 hours.

Pore water was extracted from the samples from the first 100 cm at 1 cm intervals, using the squeezing technique proposed by Shotyk and Steinmann (1994). The pH was measured immediately after extraction, using a CRISON multiprobe MM40 + meter.

The concentrations of calcium (Ca), magnesium (Mg), strontium (Sr), Pb and titanium (Ti) were determined using an Agilent 7500cx collision reaction cell inductively coupled plasma mass spectrometer (CRC-ICP-MS). Peat samples were digested by a destructive technique using a hot acid mixture of HNO_3_ and HF (Krachler 2007) in a Milestone-Ethos 1 microwave oven. An external calibration method, based on measurements of calibration standards of known concentration, was used for the conversion of the signal from the CRC-ICP-MS mass spectrometer in counts per second (cps) to a concentration in mg kg^−1^ for each element being analysed. Furthermore, continuous on-line mixing of an internal standard solution (Rh) was performed to compensate for the drift of the instrument. The conversion from cps intensities to concentrations was extrapolated from linear regressions and for all the elements R^2^ > 0.97 was obtained. All concentration values determined in this study were well above the corresponding limits of quantification (LOQ). The precision and accuracy of the analytical measurements were evaluated using two reference materials, that are NIMT/UOE/FM/001 (Yafa et al. 2004) for the solid peat phase and TMRAIN-04 (Environment Canada) for the pore water. The reproducibility was tested by digesting and analyzing four samples in triplicate (CV: < 15% for Ca, < 12% for Mg, < 3% for Pb, < 10% for Sr and < 4% for Ti).

The Pb isotopes ^206^Pb, ^207^Pb and ^208^Pb were measured using ICP-MS with an Agilent 7500cx spectrometer. The samples previously analysed for major and trace elements were diluted in order to obtain a Pb concentration < 10 μg l^−1^. The standard reference material (SRM) 981 common lead isotopic standard (IST, Gaithersburg, MD, USA) was dissolved in cold 1:1 (v/v) diluted HNO_3_ (65%), which was then diluted to a total concentration of 10 μg l^−1^. Five replicates of each sample were made, while the SRM was analysed every four samples in order to correct for mass discrimination effects. The precision of all Pb isotope ratio measurements varied between 0.01 and 0.37%.

Linear correlation (Pearson correlation coefficient) among physical and chemical parameters was calculated using SPSS v. 20.0 (IBM Corporation 2011).

### Pollen analysis

Samples were collected at a 10 cm interval for a first exploratory pollen analysis of the entire core. The resolution was subsequently improved to a 5 cm interval for the upper 125 cm of the peat sequence and then to a 2–3 cm interval for the first metre. A total of 55 samples were analysed for pollen.

For the calculation of pollen concentrations, the volume of each sample was measured and a defined amount of exotic pollen spores from *Lycopodium clavatum* tablets was added (Stockmarr 1971). The peat material was sieved and the fraction 7–150 µm chemically treated following the standard procedure for pollen analysis (Fægri and Iversen 1989). If necessary, HF treatment followed the acetolysis. The slides were stained with fuchsine and mounted in glycerine. Pollen identification and counting was carried out using an Olympus BX50 light microscope at a standard magnification of 400Χ, using 1,000Χ for critical identifications. The modern reference collection of the Botanical Institute of Innsbruck University, standard identification keys (Punt et al. 1980–2003; Fægri and Iversen 1989; Moore et al. 1991; Beug 2004) and pollen atlases (Reille 1992) were used for the identification of the pollen grains and spores. To obtain a statistically robust dataset, at least 1,000 pollen grains were counted for each pollen spectrum (Berglund 1987), excluding pollen from aquatic and wetland plants. Cerealia refers to Poaceae pollen grains larger than 45 µm and were identified by their pollen morphology (Beug 2004). Non-pollen palynomorphs and micro-charcoal particles were also quantified. NPPs were identified following van Hoeve and Hendrikse (1998), van Geel et al. (2003), using Miola (2012) for the nomenclature. Micro-charcoal fragments were identified as angular, black and opaque particles, and grouped into three size classes: < 50 µm, 50–100 µm and > 100 µm.

### Data analysis

#### Pollen data analysis

The pollen percentages were calculated using Tilia v. 2.0.41 (Grimm 2011), and expressed as the terrestrial pollen sum (TPS). TiliaGraph was used to draw the pollen diagram of the relative occurrence of selected pollen types, spores, NPPs and the influx (particles cm^−2^ yr^−1^) of micro-charcoals. The high abundance of Cyperaceae, *Pinus* and *Calluna vulgaris* on and around the peat bog could lead to their over-representation, so they were excluded from the TPS. The percentages of aquatic and wetland plants, spores, NPPs and micro-charcoal particles were calculated as a percentage of TPS. Local pollen assemblage zones (lpaz) were determined with CONISS clustering with Tilia, using a square root transformation of terrestrial pollen taxa percentages (Grimm 1987).

#### Determination of Pb enrichment factor

Pb concentrations were normalized to those of Ti, a lithogenic element considered to be immobile, conservative and resistant to chemical weathering in acidic solutions (Goldich 1938). This normalization is the basis for the calculation of the enrichment factor (EF), which represents the number of times an element is enriched in a sample compared with its abundance in the Earth’s crust (Shotyk 1996). Although criticized by some, when used with caution, the EF can help to distinguish between natural and human origins of trace metals in the environment (Shotyk et al. 2017). Here, the Pb EF (EF_Pb_) was calculated for the top 150 cm, using the formula:

1 $${\text{EF}}_{\text{Pb}} \, = \,\left( {\left[ {\text{Pb}} \right]/\left[ {\text{Ti}} \right]} \right)_{\text{sample}} /\left( {\left[ {\text{Pb}} \right]/\left[ {\text{Ti}} \right]} \right)_{\text{UCC}}$$

where [Pb] and [Ti] are the total concentrations in mg kg^−1^ of Pb and Ti respectively, in the peat sample and in the Earth’s upper continental crust (UCC) (Wedepohl 1995).

## Results

### Radiometric dating and age-depth model

The concentrations of total ^210^Pb, unsupported ^210^Pb, supported ^210^Pb and ^137^Cs in the top 40 cm of the peat core are given in Table 1. Supported ^210^Pb (^226^Ra) activity was below the level of detection in all samples above 26 cm, and only just above this level in samples below this depth. These results presumably reflect the very high organic matter content of the core. Therefore, differences between total and unsupported ^210^Pb are negligible. Unsupported ^210^Pb activity declined relatively uniformly with depth, with an apparent steepening of the gradient below 20 cm, partly due to the higher density of the deeper layers. The relatively high ^137^Cs inventory (> 3,500 Bq m^−2^) suggests that a significant fraction of it derives from fallout from the 1986 Chernobyl nuclear reactor explosion, especially the part above 14 cm. However, some irregularities are visible in the first 8 cm of the core, probably due to the biological activity of the surface vegetation (Vinichuk et al. 2010). Finally, there is no clear record of the 1963 nuclear weapons test fallout maximum. Such a migration of ^137^Cs, detected both below and above the expected depth, has been reported for several other peat bogs (for example, Mitchell et al. 1992; Gallagher et al. 2001; Zaccone et al. 2007).

**Fig. 2:**
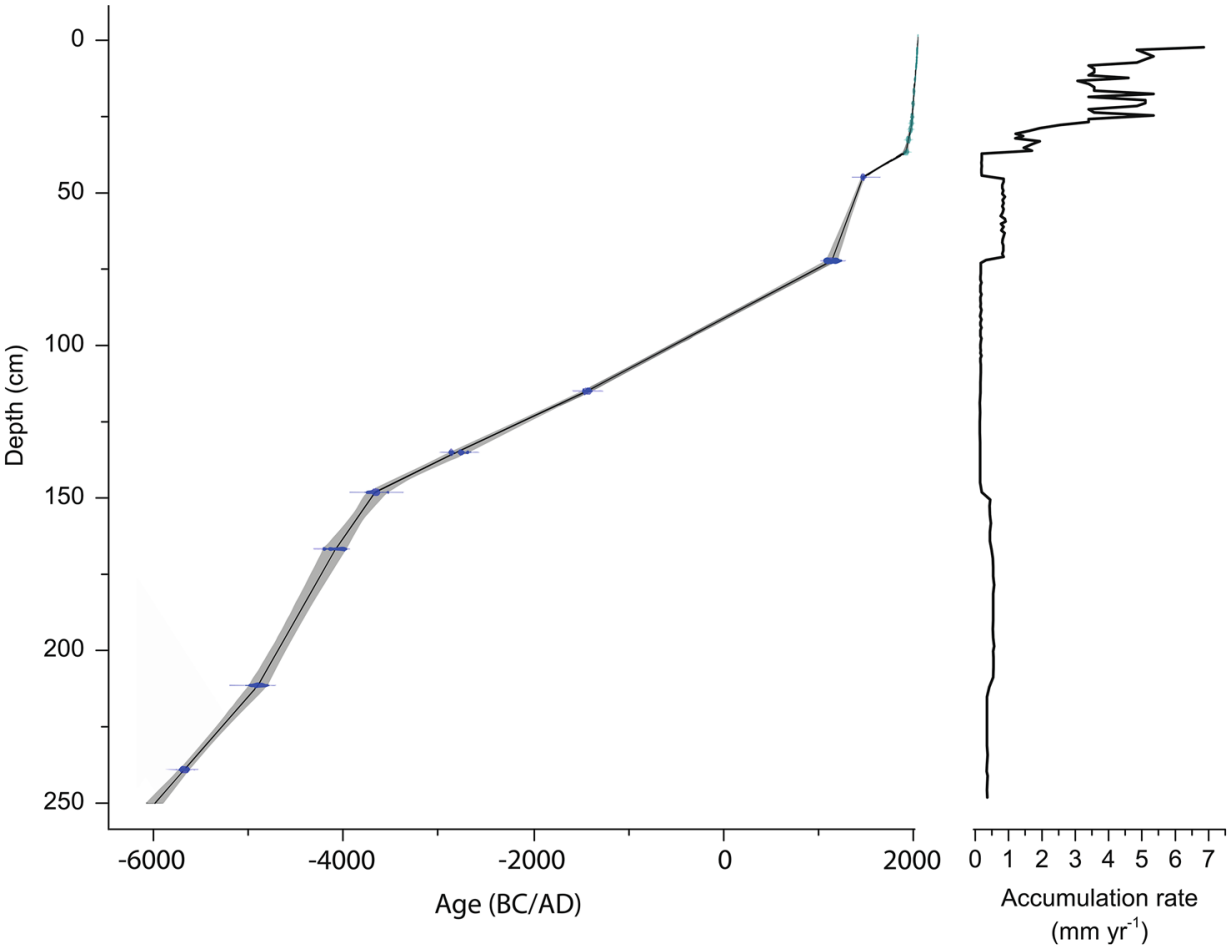
The age-depth model from Clam, based on linear interpolation of the available ^14^C, ^210^Pb and ^137^Cs measurements and the peat accumulation rate

In addition to ^210^Pb dating, eleven peat samples were radiocarbon dated (Table 2). The age-depth model was created by combining the ^210^Pb dates (n = 15) and the ^14^C ones (n = 11), which were then extrapolated down to 250 cm. The data suggest that peat accumulation at the Coltrondo bog started ca. 5950 cal bc (Fig. 2 ), covering the mid and late Holocene. The bog grew at a mean rate of 0.46 mm yr^−1^ from 5950–3750 cal bc (Fig. 2). Until ad 1030 accumulation dropped to a mean value of 0.16 mm yr^−1^, then increased to about 0.80 mm yr^−1^ for the next 340 years. After that, the accumulation rate returned to lower values (ca. 0.20 mm yr^−1^). The last 120 years are characterized by much faster growth (1.20–6.85 mm yr^−1^).

### Trophic status of the bog

**Fig. 3:**
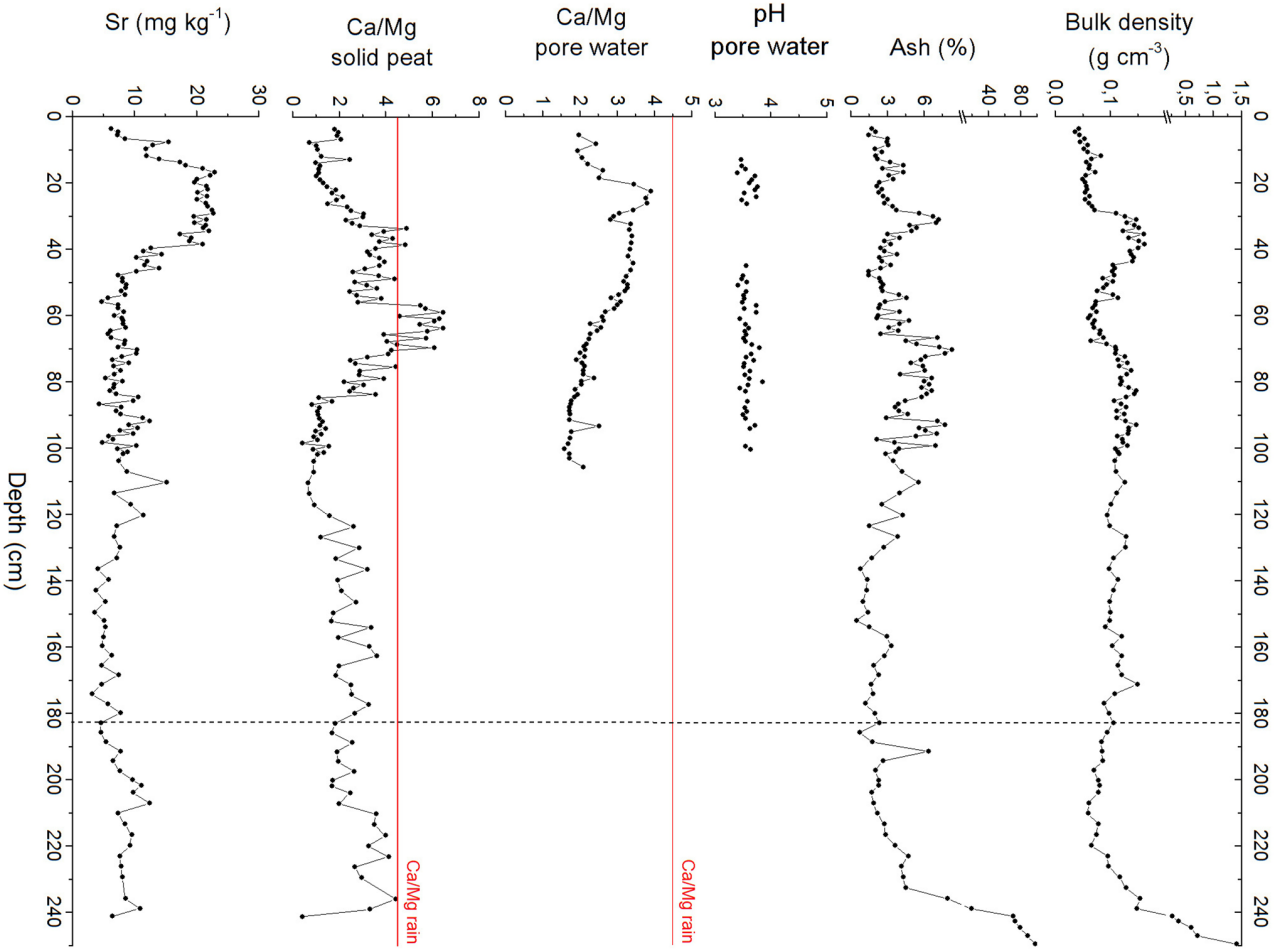
Physical and chemical properties of the bog. The red line in the graphs of Ca/Mg ratios in pore water and solid peat represents the value of the same ratio in modern rainwater, pH 4.5. The vertical dashed line indicates the probable boundary between ombrotrophic and minerotrophic peat. For some samples, it was not possible to determine the pH due to both the low water content and the high peat density. Detailed data are in ESM Table S2

A first visual inspection of the Coltrondo bog vegetation, which was mainly dominated by *Sphagnum* mosses, *Eriophorum vaginatum*, *Calluna vulgaris* and *Vaccinium microcarpum*, suggested its oligotrophic (nutrient poor) status and acidic conditions (Pignatti 1982); in fact, pore water pH values were always < 4, with a range of 3.4–3.9 (Fig. 3).

The Ca/Mg ratio in peat is generally used as an indicator for distinguishing rainwater dependent (ombrotrophic) from groundwater dependent (minerotrophic) peats, with bogs having a relatively low Ca/Mg ratio compared to fens (Shotyk 1996). The Ca/Mg molar ratio of the pore water in the first metre of the core was always < 4.5, which is the same value calculated for the local rainwater. Therefore, precipitation represents the only source of nutrients in the studied bog, thus confirming its ombrotrophic status.

The bulk density of the peat ranges between 0.04 and 0.16 g cm^−3^ (mean ± SD: 0.10 ± 0.03 g cm^−3^) down to 239 cm, without a clear trend and often consisting of poorly decomposed low density peat underlying well decomposed layers (Fig. 3); this is probably the result of environmental conditions in the past that were sometimes more conducive to rapid peat accumulation (Zaccone et al. 2018). The basal layers show a significant rise in density in the lowest 10 cm, with values up to 1.41 g cm^−3^, due to the increase of inorganic material there.

The ash content is very low throughout the profile (mean ± SD: 3.6 ± 1.8%) down to 239 cm; below this depth, a strong increase occurs, with values up to 97% at 250 cm, where the mineral substrate is reached. Ash content is positively and significantly correlated with both bulk density (*R *= 0.54, *p* < 0.01) and Ti concentration (*R *= 0.92, *p *< 0.01); this suggests that variations in ash content throughout the profile are mainly the result of changes in the rate of supply of dust particles, rather than differences in the degree of peat mineralization (Zaccone et al. 2013, 2018).

Ca and Sr concentrations show a similar trend (*R *= 0.81, *p* < 0.01), with higher values in the upper layers due to biological activity; in the lower layers below 180 cm, both elements show a moderate increase, possibly suggesting the influence of groundwater as a source (Shotyk et al. 2001; Kylander et al. 2005).

These types of proxy evidence used to evaluate the nutrient status of the Coltrondo peat bog suggest ombrotrophic conditions at least down to ca. 180 cm.

### Vegetation development

**Fig. 4:**
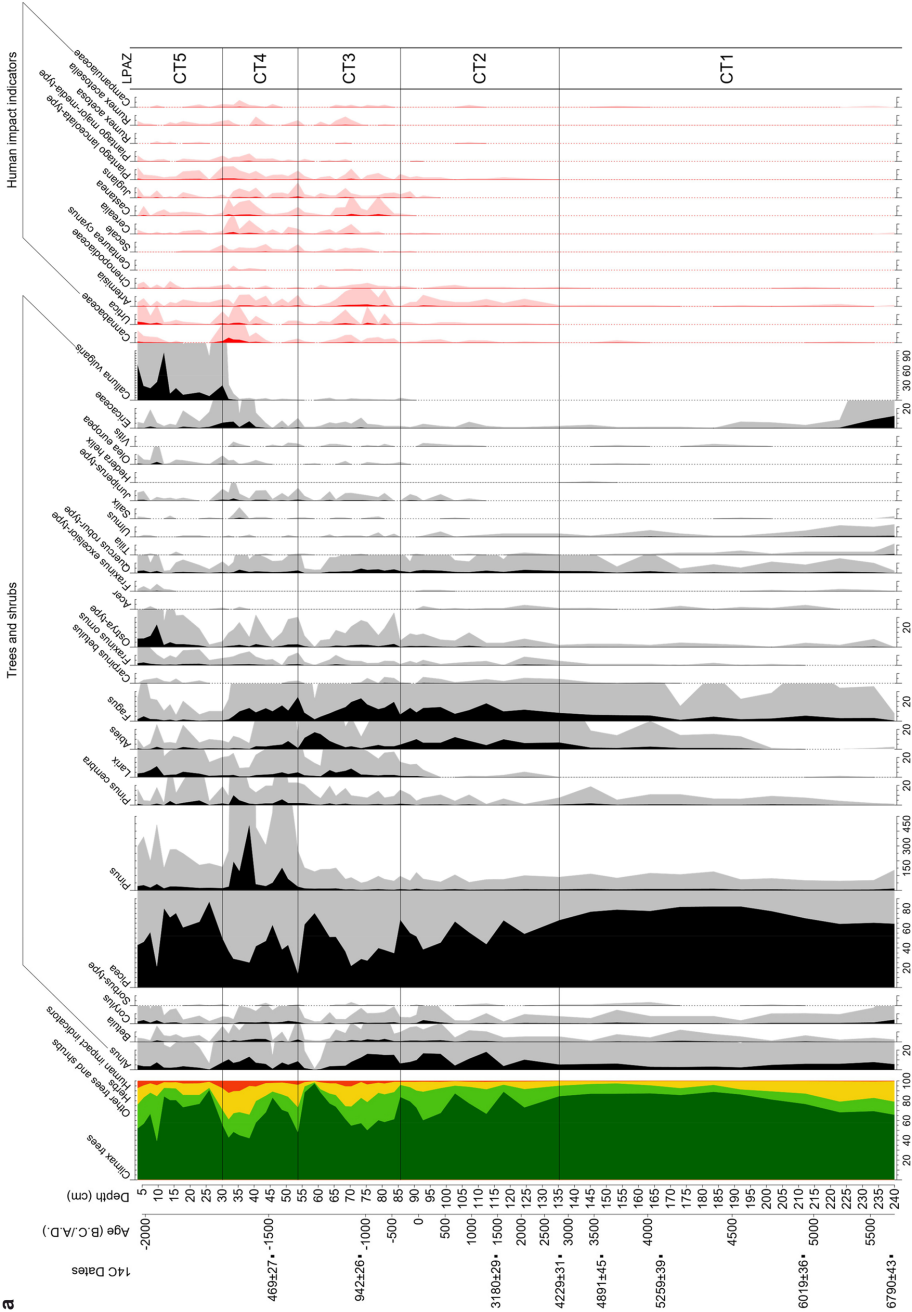

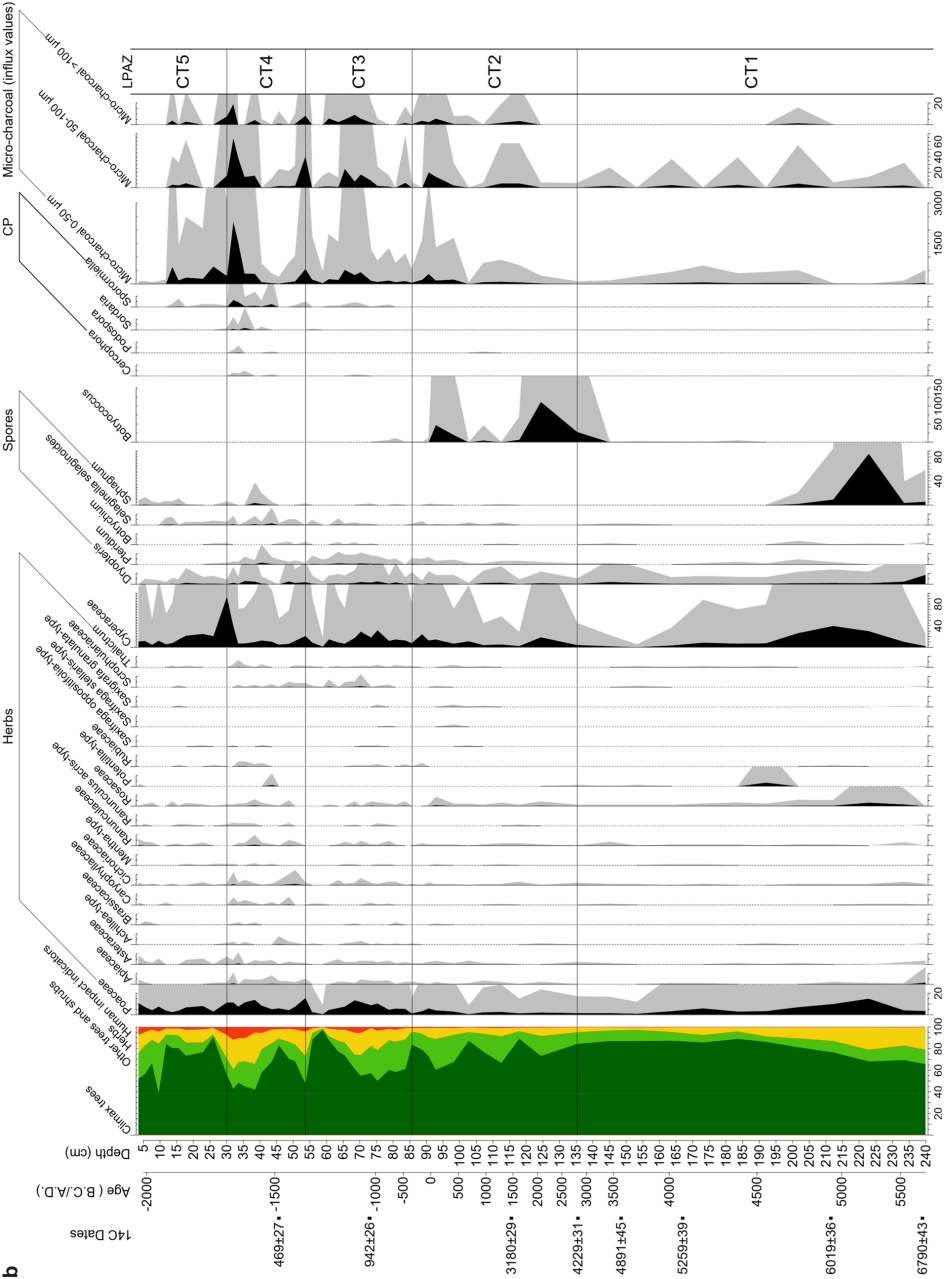
Coltrondo pollen diagram. **a** Percentage values of trees, shrubs and human indicators. The climax trees are *Picea*, *Pinus*, *Pinus cembra*, *Larix* and *Fagus*; **b** percentage values of herbs, spores, algae and coprophilous fungi (CP). Micro-charcoal particles are shown as influx values in particles cm^−2^ yr^−1^. Detailed information is given in ESM Table S3

In the pollen analysis, a total of 116 pollen and spore types as well as 34 NPPs were identified. The most significant curves representing 64 pollen and spore types and four NPPs are shown in the pollen diagram (Fig. 4). They provide the basis for the five local pollen assemblage zones (lpaz) which are discussed below and summarized in Table 3.

**Table 3:** Main characteristics of the Coltrondo local pollen assemblage zones (lpaz)

LPAZ	Depth (cm)	Age (cal bc/ad)	Archaeological period	AP/NAP % (average values)	Main pollen taxa
CT5 *Picea*-*Calluna*	30.1–3.6	ad 1935–2005	Recent	86/14	*Picea, Calluna vulgaris, Larix, Ostrya*-type, Poaceae
CT4 *Picea*-*Fagus*-*Pinus*	53.7–30.1	ad 1340–1935	Middle Ages to recent	76/24	*Picea*, *Pinus, Fagus, Abies, Larix, Alnus, Juniperus*-type, Poaceae, Cerealia*, Secale, Plantago lanceolata*-type, Cannabaceae*, Urtica, Artemisia,* Cichorioideae
CT3 *Picea*-*Fagus*-Poaceae	85.6–53.7	ad 345–1340	Late Roman period to Middle Ages	87/13	*Picea*, *Fagus*, *Abies*, *Larix*, *Alnus*, *Quercus robur*-type, *Ostrya*-type, *Juniperus*-type, Poaceae, Cerealia, *Secale*, *Plantago lanceolata*-type, *Artemisia*, *Urtica*, Cannabaceae, Chenopodiaceae
CT2 *Picea*-*Fagus*	135.5–85.6	2815 bc–ad 345	Copper Age, Bronze Age, Iron Age to Roman period	93/7	*Picea*, *Fagus*, *Abies*, *Alnus*, *Quercus robur*-type, Poaceae, *Artemisia*
CT1 *Picea*	239.9–135.5	5710–2815 bc	Neolithic	90/10	*Picea*, *Fagus*, *Alnus,* Poaceae

#### Lpaz CT1: Picea zone (5710–2815 cal bc)

The vegetation surrounding the bog in its first stages was characterized by a sparse *Picea* woodland with some *Alnus viridis* shrubs and light demanding herbs within the Poaceae and Rosaceae. This vegetational setting suggests a cool and damp climate that may have started the formation of the peat. The growth of wetland plants such as Cyperaceae and mosses (*Sphagnum* spp.) on the bog also indicates wet conditions.

From ca. 5250 cal bc onwards, the *Picea* woods spread and became denser in the lower subalpine belt (ca. 1,700–2,000 m) where the bog is located, while at even lower altitudes, in the montane zone (ca. 800–1,700 m), *Picea* formed a mixed woodland with *Fagus* and *Abies*. The occurrence of pollen of *Quercus robur*-type, *Tilia*, *Ulmus*, *Ostrya*-type, *Acer* and *Fraxinus* spp. suggests the presence of thermophilous deciduous woodland on the floor of Val Padola. The expansion of *Fagus* at about 4250 cal bc, together with the increase of *Alnus viridis* and grasses (Poaceae), suggest a wetter and cooler climate then. This climatic situation is also reflected in the simultaneous opening up of the local spruce woods. At the end of the Atlantic period, a mixed woodland of spruce, beech and fir established with the expansion of *Abies* at lower altitudes from about 3450 cal bc onwards.

#### Lpaz CT2: Picea—Fagus zone (2815 cal bc–ad 345)

At the beginning of the zone *Picea* declines, while a greater range of herbaceous taxa (Apiaceae, *Artemisia*, Asteraceae, Cannabaceae, Chenopodiaceae, Cichorioideae, *Plantago lanceolata*-type, Ranunculaceae, some Rosaceae and *Urtica)* appears. At the same time as an increase in *Alnus viridis*, Cyperaceae and *Botryococcus* which indicate wetter conditions at the site, *Abies* and *Fagus* spread at lower altitudes providing further evidence of wetter conditions above.

From 2050 cal bc*Picea* woods expanded again, while *Larix* occurred for the first time. Then, about 1650 cal bc*Picea* declined again, reaching a new minimum at 1250 cal bc, simultaneously with an expansion of *Alnus viridis* and *Fagus*, reflecting a cooler and wetter climatic phase with a shorter growing season that caused a lowering of the tree line. A similar pattern characterized another reduction in the *Picea* woods from ca. 650 to 50 cal bc, accompanied by the expansion of *Alnus viridis* and *Botryococcus*.

At about 550 cal bc, a minor increase in Poaceae and *Artemisia*, Cannabaceae, *Urtica*, Chenopodiaceae and *Plantago lanceolata*-type as well as the occurrence of *Juniperus*-type suggest moderate human activity (Behre 1981). This is the first indication of significant human impact in the area, in the early stages of the Iron Age. After 50 cal bc human presence became more evident, as shown by the start of the cereal pollen curve, first with Cerealia, followed by *Secale*, accompanied by the first occurrences of *Juglans* and *Castanea*, marking the beginning of the Roman period (Zoller 1960; Kral 1983; Kral and Carmignola 1986). Between 90 cal bc and ad 345, the pollen diagram shows an increase in climax woodland with *Picea*, *Pinus*, *P. cembra*, *Larix* and *Fagus*, and with a regeneration of the spruce woods, possibly implying more favourable climatic conditions at the site during the Roman Warm Period.

#### Lpaz CT3: Picea—Fagus—Poaceae zone (ad 345–1340)

The *Picea* woods mixed with *Abies* and *Fagus* dominated the montane belt, with a considerable presence of *Fagus* between ca. ad 350 and 900, accompanied by a severe reduction in *Picea*. In the surroundings of the mire the vegetation was more open, as indicated by the significant presence of Poaceae and other herbaceous taxa (Apiaceae, Asteraceae, Campanulaceae, Cichorioideae, Ranunculaceae, Rosaceae, Rubiaceae and Scrophulariaceae). The greater human impact both on the valley floor and near the bog is also evident from the nearly constant occurrence of *Secale* and Cerealia, as well as from the expansion of *Castanea* and *Juglans*, which were cultivated for their edible nuts. The sporadic occurrence of *Centaurea cyanus* might indicate cultivation of cereals such as *Secale* in the area surrounding the bog (Kofler and Oeggl 2010). *Olea europaea* and *Vitis* also occurred, constituting an extra-regional pollen component indicating the growing of olives and vines where the climate was Mediterranean. Their presence in the pollen spectra might be also due to either the reduction of the local vegetation cover or less local pollen production. Furthermore, other indicators of human activity such as *Artemisia*, Cannabaceae, *Urtica*, Chenopodiaceae and *Plantago lanceolata*-type are present. Also notable is the first occurrence of coprophilous fungi (*Sporormiella*; van Geel et al. 2003) that indicate animal dung and thus local human impact. The increase in bryophyte and pteridophyte spores (*Dryopteris*, *Pteridium*, *Selaginella selaginoides* and *Botrychium*) may suggest human disturbance. There is an expansion of *Larix*, possibly related to the development of larch meadows, a form of wood pasture common in the central and southern Alps (Gobet et al. 2003). Between ad 1275 and 1340, *Picea* shows a considerable decline together with a micro-charcoal peak and an increase in grasses, herbs and other human indicators, indicating more intensive human activities at high altitudes during the late Medieval Warm Period.

#### Lpaz CT4: Picea—Fagus—Pinus zone (ad 1340–ad 1935)

There was a recovery in *Picea* from ad 1340 to 1450, when a spruce woodland, mixed with *Fagus* and *Abies*, grew in the montane zone. Larch meadows developed and human activity is recorded in the pollen diagram by indicators of settlement, crops and pasture. Between ad 1450 and 1830, another opening of the woods occurred, as shown by the decrease of *Abies*, *Larix* and *Fagus*. There are considerable values of *Pinus* in this zone, possibly due to its high abundance on or around the peat bog. From about ad 1850, the highest human impact on the vegetation is detected in greater abundances of Cannabaceae and *Urtica*, indicating settlement activities, and higher percentages of *Castanea*, *Juglans*, Cerealia and *Secale*, indicating increased growing of crops. There is also evidence of pasture, mainly from *Plantago lanceolata*-type, *P. major*-*media*-type, *Rumex acetosella* and the coprophilous fungi *Sporormiella*, *Sordaria*, *Podospora* and *Cercophora* (van Geel et al. 2003). Remarkable are the high values of Cannabaceae, which might refer to hemp retting in the area near the mire. Furthermore, increased micro-charcoal influx values represent more fire activity probably from human action.

#### Lpaz CT5: Picea—Calluna zone (ad 1935–present)

Since 1935 the *Picea* woodland has started regenerating, together with a severe reduction of *Fagus*, while *Larix* has spread in the vicinity of the mire and also grows in the spruce woods. The general decrease of light-demanding herbaceous and human-related taxa is another striking feature of this zone. On the peat bog surface, *Calluna vulgaris* and *Sphagnum* mosses thrive. In the most recent samples, human indicators increase (*Artemisia*, Cannabaceae, Chenopodiaceae*, Plantago lanceolata*-type, *Urtica*) which is in agreement with the modern vegetation of the area and related to human presence, now mainly for tourism.

### Lead and lead isotopes: the evidence of human activity

#### Lead enrichment factor

**Fig. 5:**
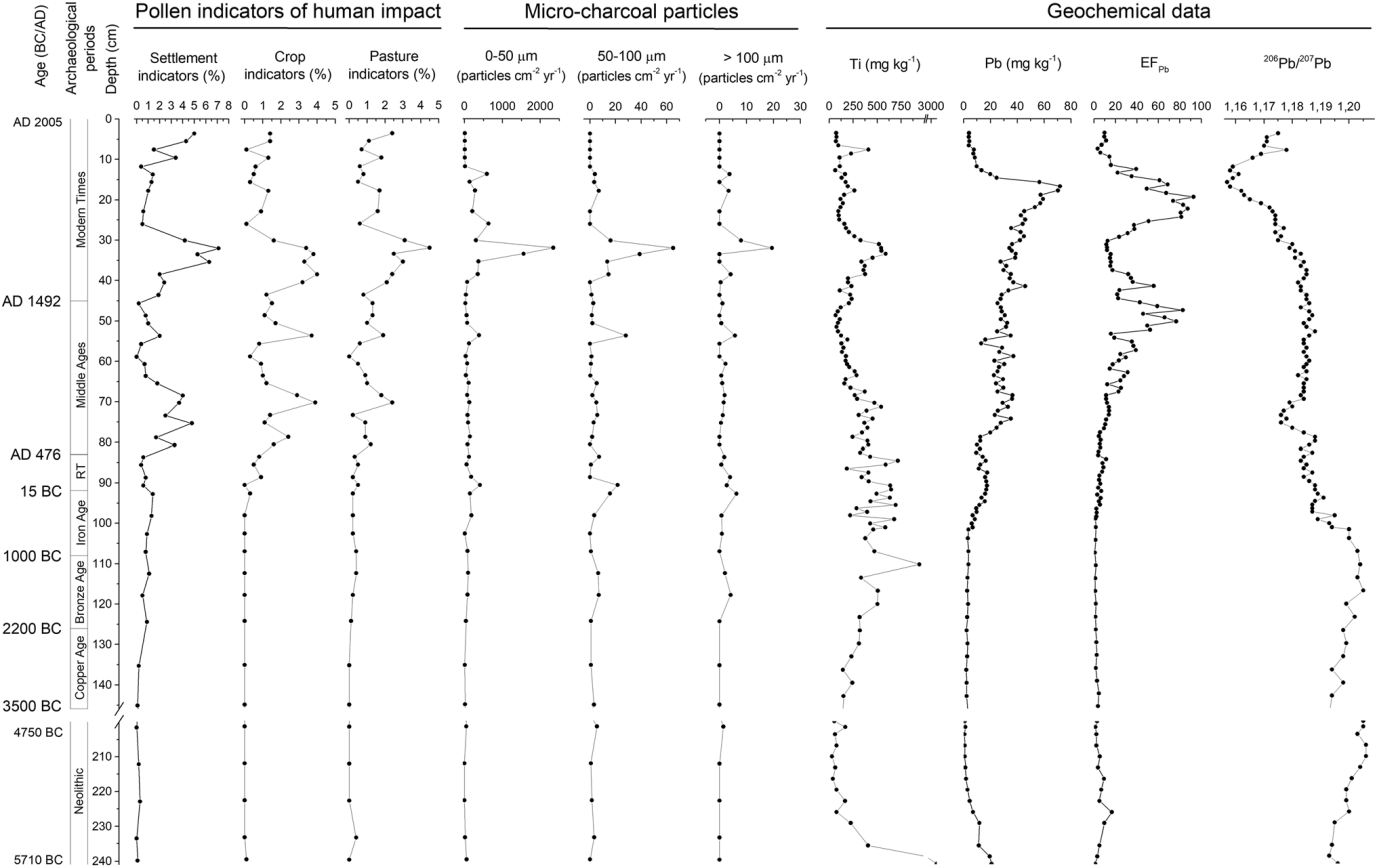
Pollen records connected with human actions, micro-charcoal influx as particles cm^2^ yr^−1^ and geochemical data. Pollen types representing human activity according to Behre (1981, 1986), Oeggl (1994) and Festi (2012). Settlement indicators are *Artemisia*, Cannabaceae, Chenopodiaceae, *Urtica*. Crops and weeds (arable farming) are *Secale*, Cerealia, *Castanea*, *Juglans*, *Centaurea cyanus*. Pasture indicators, *Aconitum*-type, Campanulaceae, Gentianaceae, *Plantago alpina*, *P. lanceolata*, *P. major*-*media*-type, *Rumex acetosa*, *Rumex acetosella*, *Trifolium*. Archaeological periods are shown according to Festi et al. (2014) (*RT* Roman times; *MT* modern times). Detailed information about the geochemical data is given in ESM Table S2

The EF_Pb_ trend is in general agreement with those reported in several other studies from different areas, showing evidence of Pb contamination dating back to the time of the Greek and then Roman civilizations, with notable episodes of intense Pb emissions during the medieval period from Ag mining in central Europe, then due to the Industrial Revolution, and finally from the introduction of leaded gasoline (Shotyk et al. 1998, 2016; Zheng et al. 2007). To start with, the EF_Pb_ is quite low from ca. 150 to 90 cm depth, from the Copper to the Iron Age, and averaging around 3.0 ± 1.7 (Fig. 5). A first significant increase in EF_Pb_ (up to 11) occurs around 87 cm, which represents the Roman period; several phases of EF_Pb_ increase are then evident through the Middle Ages (EF_Pb_ = 26 ± 20), with values up to 83 at around 49 cm, corresponding to ad 1400. The highest EF_Pb_ values (EF_Pb_ = 35 ± 27) were mainly caused by both the Industrial Revolution, the beginning of which corresponds to around 34 cm and the introduction of leaded gasoline, with a peak around the 1970s (EF_Pb_ up to 93). The sharp drop in EF_Pb_ in the top ca. 20 cm is consistent with the gradual reduction in the use of leaded gasoline (Shotyk et al. 1998) (Fig. 5).

#### Lead isotope ratios

Lead isotope ratios are widely used for distinguishing between natural and anthropogenic sources of this metal (for example, Martínez Cortizas et al. 1997; Shotyk et al. 1998, Komárek et al. 2008; Shotyk et al. 2015). The changes in the ^206^Pb/^207^Pb ratio (Fig. 5) are in general agreement with both the historical information (Cucagna 1961; Vergani 2003) and the EF_Pb_, and consistent with values recorded in other studies from across Europe (for details, see ESM Fig. S2, Table S4). From the basal peat layers up to 102 cm (ca. 650 cal bc), the ^206^Pb/^207^Pb ratio averages around 1.201 ± 0.004, which is a typical pre-anthropogenic, natural value (Klaminder et al. 2003). Between 102 to 38.5 cm (ad 1830) the values averages around 1.185 ± 0.004 and are remarkably similar to the ones associated with mining deposits in the Alpi Carniche (Carnic Alps) (Artioli et al. 2016) and in the eastern Italian Alps (Nimis et al. 2012) (ESM Fig. S2, Table S4). In particular, the values between 76.5 and 68.5 cm (1.179 ± 0.003; ad 895–1160) are quite similar to those reported by Poto (2013) for mining sites located near our bog, possibly suggesting local exploitation of mineral resources there.

The beginning of industrialization in Europe is marked by a strong decrease of the ^206^Pb/^207^Pb ratio, which reaches its lowest value of 1.157 at about 15 cm, representing the end of the 1970s, mainly due to the massive use of leaded gasoline. Its phasing out has instead resulted in the recent small increase in the ^206^Pb/^207^Pb ratio (Farmer et al. 2005). This trend is visible in the nearby bog of Danta di Cadore (Poto 2013) and has been noted in many other studies carried out on European peat bogs (for example, Shotyk et al. 1998, 2003; Bindler et al. 2004; Farmer et al. 2005; Kylander et al. 2005).

## Discussion

### Neolithic, Copper and Bronze Age

**Fig. 6:**
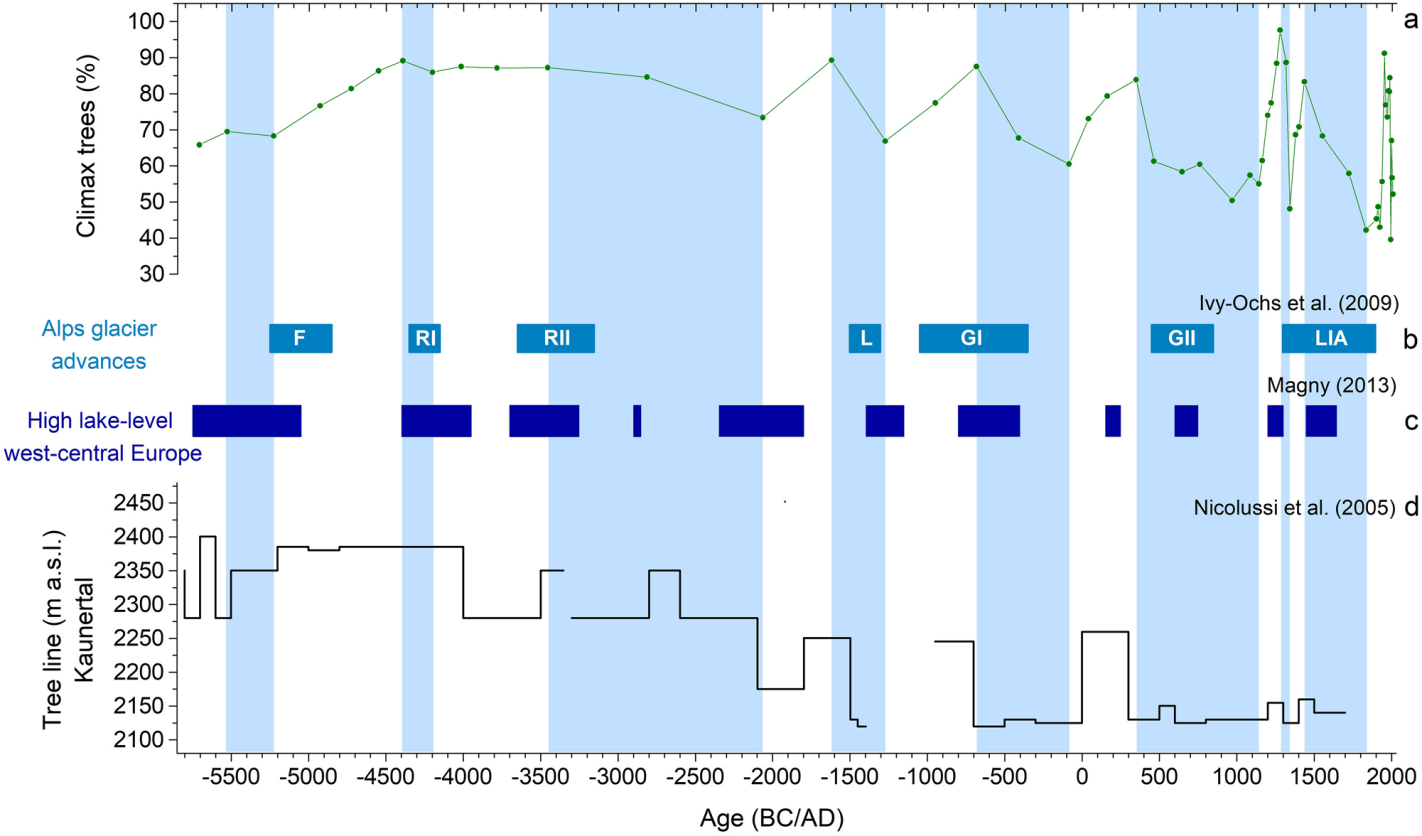
Comparison of **a** the Coltrondo peat bog climax community with other palaeoclimate records; **b** main Alpine glacier advances during the cold oscillations Frosnitz (F), Rotmoos I and II (RI and RII), Löbben (L), Göschener I and II (GI and GII), Little Ice Age (LIA) (Ivy-Ochs et al. 2009); **c** high lake levels in western-central Europe (Magny 2013); **d** tree line changes in Kaunertal, eastern Alps (Nicolussi et al. 2005). Blue bands indicate colder phases noted in the pollen diagram

The abundant occurrence of Cyperaceae and *Sphagnum* in the basal layers of the core suggests that the Coltrondo mire is a genuine (ombotrophic) raised bog, while the presence of *Picea* together with light demanding taxa, such as Poaceae and Rosaceae, suggests that a sparse woodland grew nearby until about 5250 cal bc. The start of peat growth and the type of vegetation in this period both seem to be related to a period of cooler and wetter climate in the eastern Alps, the Frosnitz deterioration (Fig. 6; Patzelt 1977). This climatic change caused either a lowering of the tree line and/or a reduction in pollen production caused by a shorter, cooler, growing season (Kofler et al. 2005), while grasses (Poaceae) and other herbs (Rosaceae) increase, indicating a thinning of the spruce woods at high altitudes.

This event is also recorded in other pollen records from the Alps (such as Seiwald 1980, Kral 1986b, Wick and Tinner 1997, Haas et al. 1998, Kofler et al. 2005) (for the eastern alpine sites, see ESM Fig. S1, Table S1). The advance of the Gepatschferner glacier in the eastern Alps (Nicolussi and Patzelt 2000) and the lake level fluctuations studied by Magny (2013) in the central Alps are also connected with this climatic oscillation (Fig. 6). During this time, total solar irradiation was at its lowest level of the Holocene (Steinhilber et al. 2009).

The subsequent thickening of the woods shown by the maximum of tree pollen reached at about 4,550 cal bc is possibly related to the Holocene Thermal Optimum (ca. 11,000–5,000 cal bp/9050–3050 cal bc; Renssen et al. 2009), a period characterized by higher summer temperatures in the Northern Hemisphere (Wanner et al. 2011). Other sites in the eastern and central Alps also show the development of dense woodlands (for example, Seiwald 1980; Kral 1986b; Oeggl and Wahlmüller 1994; Wick and Tinner 1997; Stumböck 2000; Burga and Egloff 2001; Pini 2002; Heiss et al. 2005). At that time, the tree line in the eastern Alps reached its maximum height for the Holocene (Fig. 6; Oeggl and Wahlmüller 1994; Wick and Tinner 1997; Nicolussi et al. 2005). During this period, however, the climate was not stable and several variations occurred, such as the Frosnitz event, which caused a temporary lowering of the tree line and other effects on the vegetation. The expansion of *Fagus* in the mountain zone at about 4250 cal bc, accompanied by *Alnus viridis* and an increase in grasses (Poaceae), together with a reduction of *Picea*, suggests a response to a wetter and colder climate, leading to a more open vegetation cover. The following expansion of more oceanic trees, *Fagus* accompanied by *Abies*, around 3450 cal bc and the reduction of the spruce woods, possibly due to a lowering of the tree line or a shorter growing season, suggests another return to a wetter and cooler climatic phase. These two successive climatic variations correspond to two cooling events, Rotmoos I and Rotmoos II (Fig. 6), observed for the first time by Bortenschlager (1970) in the pollen record from Rotmoos, a mire in the Ötztal Alps, Austria. Other pieces of pollen evidence for these cold phases are given by Seiwald (1980), Wick and Tinner (1997), Haas et al. (1998), Burga and Egloff (2001) and Kofler et al. (2005). During the Rotmoos I oscillation (4350–4150 cal bc), the eastern alpine glaciers advanced (Nicolussi and Patzelt 2000), lake levels were higher in the central Alps (Magny 2013) and a significant lowering of the tree line by about 200 m is recorded in the Kaunertal (Kofler et al. 2005; Nicolussi et al. 2005) (Fig. 6). This regressive phase also corresponds with the Piora I oscillation, which was first recorded in the Swiss Alps by Zoller (1960). The Rotmoos II oscillation (3650–3150 cal bc) (Bortenschlager 1970; Patzelt 1977) affected the entire alpine region (Baroni and Orombelli 1996; Wick and Tinner 1997; Haas et al. 1998; Nicolussi and Patzelt 2000) and elsewhere (Mayewski et al. 1997; Solomina et al. 2015).

From ca. 3450 cal bc*Picea* decreased its altitudinal range. The significant presence of *Botryococcus* in the bog suggests moister climatic conditions, also indicated by an expansion of *Alnus viridis*. These changes in the trees represent the start of the Neoglacial period in the eastern Italian Alps (Deline and Orombelli 2005; Magny et al. 2006; Simonneau et al. 2014). This was characterized by lower summer temperatures from reduced sunlight during the Northern Hemisphere summer, mainly caused by changes in orbital forcing (Kofler et al. 2005; Renssen et al. 2006; Wanner et al. 2011; Solomina et al. 2015).

Since the beginning of the Bronze Age at ca. 2250 cal bc, the pollen record shows some indicators of human action, such as *Artemisia*, Cannabaceae, Chenopodiaceae, *Urtica* and *Plantago lanceolata*-type, possibly indicating moderate human activity. Nevertheless, the low percentages of these indicators and also low micro-charcoal influx values (cf. Leys et al. 2014) at this point (Fig. 5), suggest that the origin of this evidence is more regional than local. In the nearby Val Pusteria, human settlements are known on the valley floor from the Neolithic and higher up since the Bronze Age (Lunz 1977). Anyway, there is no corresponding archaeological evidence from the Cadore area up to now to confirm any evidence of local human activity (Collodo 1988).

Between 1650 and 1250 cal bc there was an opening of the spruce wood and an expansion of *Alnus*, this possibly a sign of another cooler phase with a shorter cold/wet growing season that caused a lowering of the tree line again. This phase might be related to the Löbben oscillation, a period of cool and damp climate recorded in the Alps by the advance of numerous glaciers (Ivy-Ochs et al. 2009), higher lake levels (Magny 2013), the lowering of the tree line (Fig. 6; Nicolussi et al. 2005) and first observed in the eastern Alps by Patzelt and Bortenschlager (1973). Other pollen evidence for a cool and damp phase during this period is provided by Seiwald (1980), Haas et al. (1998), Schmidt et al. (2007) and Schneider et al. (2010). Cooler and moister conditions were also widespread elsewhere in the Northern Hemisphere (Bond et al. 2001; Solomina et al. 2015).

### Iron Age and Roman period

The reduction in *Picea* between ca. 650 and 50 cal bc represents a climatic event corresponding to Göschener I (Fig. 6), first detected from pollen analysis by Zoller et al. (1966) and also noted in many other regions of the Alps by different proxies (for example, Haas et al. 1998; Holzhauser et al. 2005; Ivy-Ochs et al. 2009; Magny 2013). This vegetation change, of regional dimension, has also been detected near the Coltrondo site (Kral 1986a, 1991) and the main Alpine ridge (Oeggl and Wahlmüller 1994) (ESM Fig. S1, Table S1). The transition between the Sub-Boreal and Sub-Atlantic is conventionally placed in this period, ca. 550 cal bc, and characterized by a cooler and wetter climate in northern Europe (Wanner et al. 2008). Since the Iron Age, human presence in the valley starts to be evident in the pollen record, and about 50 cal bc, during the Roman period, the occurrence of Cerealia, *Secale*, *Juglans* and *Castanea* shows the expansion of human activity. While the extent of human impact at that time in the Comelico area does not represent an addition to current knowledge (Pirazzini et al. 2014), this is the first study to suggest earlier settlement in the area starting from 650 cal bc, the Iron Age. Human impact is also reflected in the geochemical data, which indicate a slight increase of the EF_Pb_ during the Roman period (up to 11), as well as a decrease in the ^206^Pb/^207^Pb ratio (down to 1.183; Fig. 5). It is known that Pb–Zn ores were extracted in the Comelico area during the Middle Ages, at Santo Stefano di Cadore (Salafossa mine, located 16 km from the Coltrondo bog), at Auronzo (Argentiera mine, about 12 km away) and in Valle Inferna (Argentiera mine, about 36 km away) (Fig. 1; De Lorenzo 1999), but no earlier signs of ore extraction are known up to now. However, as reported by historical studies (Cucagna 1961; De Lorenzo 1999; Vergani 2003), local mining activity during the Roman period is likely, and its effects may have been recorded in the peat bog. During the Roman period, Pb was mined all around Europe, as shown by many palaeoenvironmental studies carried out mainly on lakes and mires in Spain (Martínez Cortizas et al. 1997, 2002; Monna et al. 2004a; Kylander et al. 2005), in the British Isles (Le Roux et al. 2004; Mighall et al. 2009, 2014; Küttner et al. 2014), in Switzerland (Shotyk 1996; Shotyk et al. 1998), in France (Monna et al. 2004b) and in Sweden (Brännvall et al. 1997, 1999; Klaminder et al. 2003).

The evidence for regeneration of the woodlands between 90 cal bc and ad 345 possibly implies an improvement in the climate, because indicators of human activities are constantly present and rising apart from *Artemisia*. Geochemical proxies studied in Austrian alpine lakes also support a warmer phase during this period (Schmidt et al. 2007). Roman times are indeed characterized by high total solar irradiation (Steinhilber et al. 2009) and warm climatic conditions in Europe and in the Northern Hemisphere (Bond et al. 2001), and by the absence of glacial advances in the eastern alpine region (Nicolussi and Patzelt 2000).

### The Middle Ages and Modern times

During the Middle Ages, the pollen record indicates higher human impact. The regular occurrence of *Secale* and other cereals, as well as the expansion of *Castanea* and *Juglans*, suggest that the fertile soils in the valley were being cultivated, whereas the increase of *Larix* at higher altitudes possibly indicates the development of larch meadows due to grazing there (Gobet et al. 2003), as also suggested by pollen types indicative of pasture and the first occurrence of coprophilous fungi. From ca. ad 700 a gradual increase in the EF_Pb_, coupled with a ^206^Pb/^207^Pb ratio characteristic of mining sites in the same area (1.166–1.188; Artioli et al. 2016), and in combination with the decline of *Picea*, may suggest local mining, well in agreement with the signs of increasing human impact in the area, as seen in the pollen data (Fig. 5). In addition to arable and pastoral farming, ore deposits were an important resource for the population in these severe mountain environments, as shown by the presence of *laudi* (written rules) for the proper management of these activities (Rosolo 1982; Cesco Frare 2011). Such deposits mainly consisted of Fe, Cu, Pb and Zn ores. Between ad 900 and 1200, population increase and economic growth characterize Italy and the whole Europe (Brännvall et al. 1999). This is also reflected in the Coltrondo peat bog, which indicates further human impact, with the flourishing of farming and mining activities. Between the 11th and 12th centuries, according to Leidlmair et al. (2002), the Comelico area was inhabited up to 1,400 m and thriving metallurgical activities increased during this period in the Cadore area (cf. De Lorenzo 1999; Vergani 2003). The local situation well reflects the general trend typical of Europe for that period (Brännvall et al. 1999; Breitenlechner et al. 2010; Mighall et al. 2014; Viehweider et al. 2015). Mining activity also had an impact on the vegetation, because of the use of wood, mainly beech, for making the charcoal used in smelters and forges (De Lorenzo 1999). The curve of *Fagus* indeed starts to decrease in this period.

From ca. ad 900 until about the end of the 13th century, a regeneration of the spruce woods is evident, mixed with firs, possibly reflecting the more favourable climatic conditions of the Medieval Warm Period. This is also supported by a higher peat accumulation rate of 0.82 ± 0.10 mm yr^−1^ between 47 and 73 cm (Fig. 2). This phase lasted between ca. ad 900 and 1300 (Holzhauser et al. 2005; Kress et al. 2014) when the *Picea* woods reached their maximum, as shown by the percentages (ca. ad 1270). Human indicators almost disappear from the pollen record, probably because the densely wooded area masked the human signals.

The sharp decline of the woodlands between the 13th and 14th century as well as the increase in micro-charcoal particles and pollen indicators of human activity (Fig. 5) reflect clearance of woodland for cultivated areas as well for timber, which from the 14th century started to have a role in the mountain economy (Leidlmair et al. 2002). Cesco Frare (2008) indeed reports a strong reduction of conifers such as *Picea* in favour of beech in the Cadore area during the 15th century due to the high demand for spruce from the Republic of Venice. Moreover, the EF_Pb_ suggests high human pressure, reaching its highest values in the Middle Ages, probably in relation to the extensive mining activities in the Cadore area, in which the three mines, Argentiera of Valle Inferna, Auronzo and Salafossa were active, even if only intermittently, in these centuries (Vergani 2003) and also all over Europe (Brännvall et al. 1999; Vergani 2003). The subsequent decrease of the EF_Pb_, taking into account radiocarbon uncertainties, may show the general decrease in mining activities throughout Europe and also in the Cadore area (Vergani 2003) that followed the discovery of America and the consequent importation of metals from there. There is a new peak of lead in the 17th century, followed by a decrease that may be ascribed to the abandonment of several mines with the decline of the Republic of Venice.

The signs of reduction in woodland between 1450 and ca. 1830 reflect the well-known economic importance of timber for the Republic of Venice between the 15th and 18th centuries, and also later. This decrease in the climax community vegetation is accompanied by a drop in the accumulation rate, 0.18 mm yr^−1^ between 39 and 46 cm (Fig. 2); the severe reduction in the climax trees may be interpreted as a combination of an unfavourable climatic phase and human disturbances.

During the second half of the 19th century and the first decades of the 20th, the pollen spectra record strong human impact on the vegetation, with the highest occurrence of settlement, crops and pasture indicators, accompanied by the highest values of total micro-charcoal particles, with 2,425 particles cm^−2^ yr^−1^ around 32 cm around 1920, possibly representing increased fire activity due to human action (Fig. 5). This is in agreement with the evidence of an increasing population in the Comelico area and with the historical records of high altitude farming up to 1,400 m in this period (Leidlmair et al. 2002). The EF_Pb_ at 39–31 cm, between 1830 and 1930, averages around 14 ± 2; however, the comparatively higher Ti and ash concentrations (454 ± 97 mg kg^−1^ and 4.7 ± 1.6%, respectively) recorded during this period (Figs. 3, 5) seem to suggest that most of the Pb reaching the bog was in the form of mineral dust (Pb vs. Ti, *R*^*2*^= 0.75, *p *= 0.02), probably caused by land use changes such as mining and farming. Studying the peat bog of Danta di Cadore, Poto (2013) observed an enrichment in Pb, Ag and cadmium (Cd) during the same period, probably related to the activity at the Argentiera of Auronzo and Salafossa mines.

Since 1935 a thickening of the spruce woods, mixed mainly with *Larix* and a general decrease of light-demanding and human-related herbaceous taxa, is seen. The peat bog was characterized by abundant *Calluna vulgaris* and by a very high accumulation rate (Fig. 2). The latter is probably related to the ongoing climatic warming and therefore to more suitable climatic conditions for the growth of *Sphagnum* mosses. The general trend of the vegetation mainly reflects the gradual abandonment of the area by humans. As stated by Leidlmair et al. (2002), after the Second World War there was a short demographic expansion until economic recovery stopped it and caused a rural depopulation. The post-war industrialization period is clearly distinguishable in the peat bog profile by both very high EF_Pb_ values found between 1930 and 1986 and low values of the ^206^Pb/^207^Pb ratio due to the introduction of leaded gasoline. The subsequent increase of the ^206^Pb/^207^Pb ratios is ascribable to the introduction of unleaded gasoline (Fig. 5). Nowadays, the Comelico area is devoted to industrial activities, mainly in optics, and to mountain tourism.

## Conclusions

The study of the Coltrondo peat bog has revealed its potential as a palaeoclimatic and palaeoenvironmental archive. The chronology obtained, covering the last 7,900 years, allows the interpretation of the data in an accurate time framework, providing new insights into the climatic and human history of the Comelico area during the Holocene. The major vegetation changes recorded by the bog before human settlement in the area are connected with the main climatic oscillations known for the Holocene in the Alps, such as the Frosnitz event, the Rotmoos I and II oscillations, the transition to the Neoglacial, the Löbben event and Göschener I. From ca. 50 cal bc, human activities started to have a noticeable effect on the vegetation, making climatic and human factors difficult to disentangle: the Roman Warm Period, the Medieval Warm Period, and the current global warming are represented by changes to the vegetation, but the amplitude of the natural variability of the climate may not be clearly distinguishable from human-related changes. The pollen and Pb records provide a precise reconstruction of human history in the area, giving important information since the first settlement. The presence of people in the valley is suggested since the Iron Age, with more intense activity starting from 50 cal bc during the Roman period, as shown by the occurrence of pollen such as Cerealia, *Secale*, *Juglans* and *Castanea*. The physical and geochemical proxy evidence which is complementary to the pollen data adds important information about human activities in the area. Mining activity is indicated since the Roman period, and reached its peak during the Middle Ages, while industrial activities are registered from the second half of the 19th century. The investigation of different lines of proxy evidence allows us to unravel various aspects of the Comelico area in the past, giving new insights on the climatic and human history of the area, still scarcely investigated.

## Electronic supplementary material

Below is the link to the electronic supplementary material.

Supplementary material 1 (PDF 1,062 kb)
