# Supplementary material for: Holocene vegetation history and human impact in the eastern Italian Alps: a multi-proxy study on the Coltrondo peat bog, Comelico Superiore, Italy
Source: Veg Hist Archaeobot. 2019 Oct 1;29(4):407–26. doi: 10.1007/s00334-019-00749-y (PMC7319406; doi:10.1007/s00334-019-00749-y)
Supplement: Supplementary file 1 — Supplementary material 1 (PDF 1,062 kb) [file 334_2019_749_MOESM1_ESM.pdf]

Holocene vegetation history and human impact in the eastern Italian Alps: a multi-proxy study on the Coltrondo peat bog, Comelico Superiore, Italy

Michela Segnana*, Klaus Oeggl, Luisa Poto, Jacopo Gabrieli, Daniela Festi, Werner Kofler, Piergiorgio Cesco Frare, Claudio Zaccone, Carlo Barbante

* Corresponding author: Michela Segnana (michela.segnana@gmail.com)

Supplementary material

Geological and geomorphological features of the area

The area is characterized by the Paleozoic crystalline basement, directly overlaid by the Lower Permian volcanic sequence. The Coltrondo peat bog rests directly over the Mid Permian Sesto (Sexten) Conglomerate (Dal Cin 1972) and the Upper Permian red beds (Val Gardena Sandstone) that were deposited in a semi-arid setting of alluvial fans, braided streams and meandering rivers (Casati et al. 1982). These lithological features entail a strong water retention and a low permeability, favouring the formation of peatlands and wetlands. The rounded shapes of the landscape are due to the easy erodibility of the rocks and mainly caused by the activities of the Piave glacier during the Last Glacial Maximum. This area is characterized by ore-bearing Devonian-Dinantian carbonates; the mineralizations are strata bound to a carbonate sequence and mainly consist of sulphides, barite and fluorite, associated to gangue that varies regionally (Brigo et al. 1988, 2001).

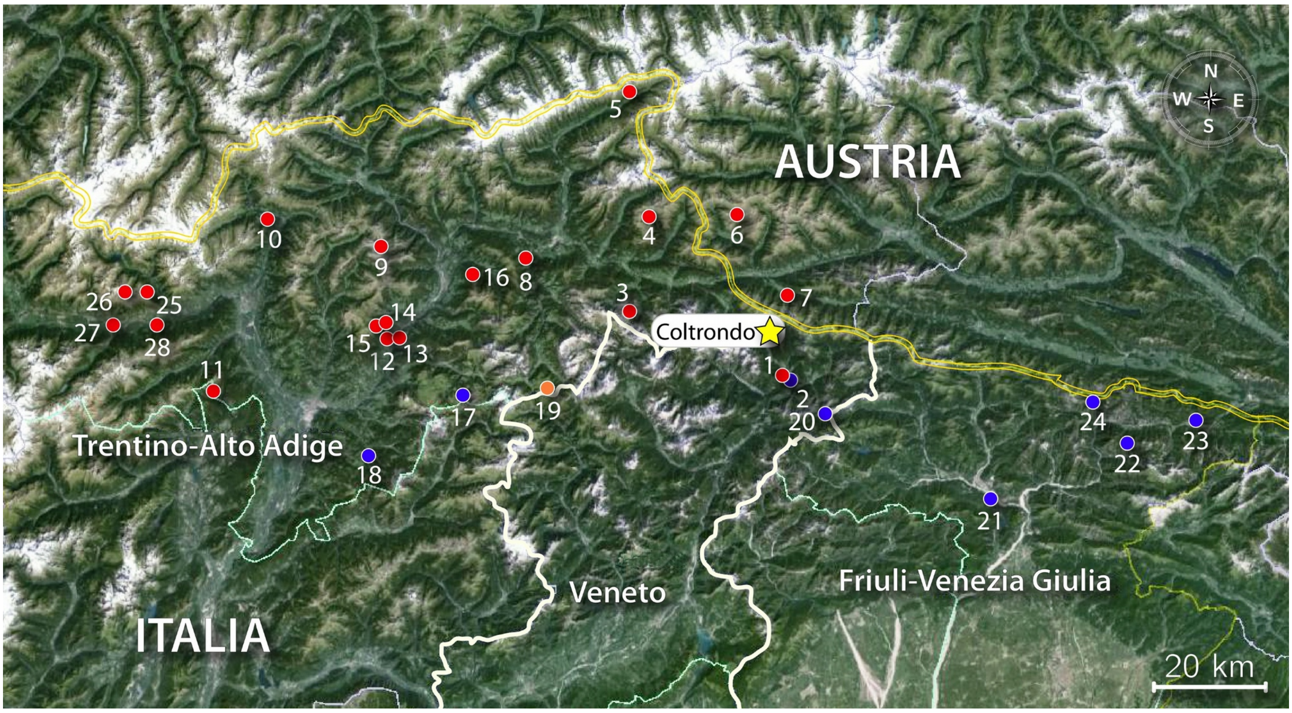

Supplementary Figure S1 – Map reporting the Coltrondo site and other palynological studies carried out in the investigation area. Yellow star = Coltrondo peat bog; red dots = studies relying on radiocarbon dating; blue dots = studies not relying on radiocarbon dating; orange dots = studies not covering the interval of interest for this study. See Table S1 for details.

Supplementary Table S1 – List of palynological studies carried out in north-eastern Italy and Austria, i.e. in sites next to the Coltrondo area. Y = radiocarbon dates (in brackets the number of ^{14}C dates; * = the study uses some ^{14}C dates from other studies), N = no radiocarbon dates. Moreover, the interval of time investigated in these studies is checked: Y = it covers the range of the Coltrondo peat bog core, N = out of the Coltrondo peat bog range.

	Site	Altitude (m a.s.l.)	Radiocarbon dates	Interval of time	Reference
1	Lago di Sant'Anna	1390	Y (2)	Y	(Kral 1986a)
2	Danta mire	1420	N	Y	(Kral 1986a)
2			Y	N	(Poto 2013)
3	Lake Braies	1492	Y (4)	Y	(Schneider et al. 2010)
4	Rasner Möser	1100	Y (2)	Y	(Kral 1991)
5	Wieser-Werfer	2075	Y (2)	Y	(Kral 1991)
6	Hirschbichl	2140	Y (3*)	Y	(Oeggl and Wahlmüller 1994)
7	Kartitscher Moor	1520	Y (2)	Y	(Kral 1988)
8	Astalm	1955	Y (1)	Y	(Burga and Egloff 2001)
9	Penser Joch	2230	Y (2)	Y	(Burga and Egloff 2001)
10	Kurzmoos	1820	Y (10)	Y	(Stumböck 2000)
11	Totenmoos	1718	Y (10)	Y	(Heiss et al. 2005)
12	Dura-Moor	2080	Y (6)	Y	(Seiwald 1980)
13	Rinderplatz	1780	Y (4)	Y	(Seiwald 1980)
14	Malschötscher Hotter	2050	Y (4*)	Y	(Seiwald 1980)
15	Schwarzsee	2033	Y (2)	Y	(Seiwald 1980)
16	Sommersüß	870	Y (5)	Y	(Seiwald 1980)
17	Großes Moos	1880	N	Y	(Kral and Carmignola 1986)
18	Biotop Wölflmoor	1295	N	Y	(Kral 1983)
19	Pescosta palaeolake	1521	Y	N	(Borgatti et al. 2007)
20	Malga Varmost	1480	N	Y	(Kral 1982)
21	Cavazzo-Vuarbes	270	N	Y	(Kral 1982)
22	Laghetto di Somdogna	1442	N	Y	(Kral 1982)
23	Malga di Lussari	1554	N	Y	(Kral 1982)
24	Passo di Pramollo	1551	N	Y	(Kral 1982)
25	Lake Vernagt	1610	Y (7)	Y	(Festi et al. 2014)
26	Lagaun mire	2180	Y (9)	Y	(Festi et al. 2014)
27	Schwarzboden mire	2150	Y (11)	Y	(Festi et al. 2014)
28	Penaud mire	2330	Y (4)	Y	(Festi et al. 2014)

Supplementary Table S2 – Physical and chemical data.

Average depth (cm)	Bulk density (g cm^{-3})	Ash (%)	pH pore water	Ca (mg kg^{-1})	Mg (mg kg^{-1})	Sr (mg kg^{-1})	Ti (mg kg^{-1})	Pb (mg kg^{-1})	EF_{Pb}
**3.6**	0.04	1.7		1810.2	1006.5	6.2	74.3	4.0	9.8
**4.5**	0.04	2.0		1706.9	874.6	7.3	75.2	3.9	9.5
**5.5**	0.04	1.4		1550.0	813.5	7.2	68.6	4.2	11.2
**6.6**	0.05	3.0		1500.1	730.8	8.5	96.6	3.8	7.1
**7.6**	0.04	2.9		1667.2	2347.6	15.4	409.7	7.6	3.4
**8.6**	0.06	3.1		1465.9	1463.8	12.9	227.5	7.3	5.9
**9.6**	0.05	2.0		1428.0	1355.2	11.8	106.9	8.4	14.4
**11.8**	0.08	2.0		1389.3	1147.4	11.9	112.0	9.7	15.8
**12.8**	0.07	2.2	3.46	1415.1	579.3	14.0	62.1	13.3	39.2
**13.7**	0.06	3.2		1551.8	1593.0	17.3	165.0	19.8	22.0
**14.6**	0.06	4.3	3.48	1539.4	1313.6	18.2	129.8	24.7	35.0
**15.6**	0.06	2.6	3.54	1802.9	1604.1	21.0	169.9	56.6	61.1
**16.7**	0.07	4.3	3.4	1812.8	1618.6	22.9	191.9	71.9	68.6
**17.8**	0.05	3.1	3.71	1657.8	1658.0	22.1	261.5	70.2	49.2
**18.9**	0.05	3.5	3.65	1601.2	1387.3	20.0	156.6	57.4	67.2
**19.9**	0.05	2.3	3.61	1543.1	1181.6	19.6	117.0	59.2	92.8
**20.9**	0.06	2.1	3.76	1725.1	1170.7	21.6	142.1	57.2	73.7
**21.9**	0.06	2.5	3.71	1733.0	945.0	21.7	117.1	53.1	83.1
**22.9**	0.05	2.3	3.52	1681.7	995.9	20.1	95.1	45.2	87.2
**23.9**	0.06	2.6	3.74	1732.2	811.8	21.7	96.8	42.7	80.9
**25.0**	0.05	3.0	3.48	1627.1	872.2	20.0	103.1	45.8	81.5
**26.0**	0.06	2.7	3.56	1669.3	1126.5	21.5	159.3	44.1	50.7
**27.1**	0.07	3.4		2201.2	947.4	21.8	175.2	35.5	37.2
**28.1**	0.07	3.7		2055.1	822.1	22.5	206.8	42.4	37.6
**29.1**	0.11	5.6		1791.7	589.5	22.7	263.1	45.0	31.4
**30.1**	0.13	6.7		1392.4	463.3	19.5	326.5	42.0	23.6
**31.0**	0.15	7.2		1174.9	514.8	21.5	520.9	36.2	12.7
**32.0**	0.13	6.9		1096.8	431.0	19.7	542.4	34.1	11.5
**32.7**	0.14	4.8		1239.8	430.0	21.5	543.5	35.8	12.1
**33.5**	0.15	5.4		1244.1	254.2	21.0	589.6	38.8	12.1
**34.4**	0.12	5.0		1380.8	354.4	22.0	452.0	38.3	15.6
**35.4**	0.16	3.0		1149.1	340.3	17.3	335.2	27.6	15.1
**36.5**	0.13	4.0		1262.4	294.7	19.1	370.6	31.8	15.8
**37.5**	0.15	2.7		1215.2	326.8	18.8	359.4	29.8	15.2
**38.5**	0.16	3.2		1468.1	304.4	20.9	373.3	35.2	17.3
**39.5**	0.15	2.4		837.7	236.4	12.7	196.2	34.3	32.0
**40.5**	0.13	2.7		782.1	243.5	11.4	196.7	37.0	34.5
**41.5**	0.14	3.7		927.1	281.0	14.3	232.4	45.8	36.1
**42.5**	0.14	2.3		866.6	232.7	10.2	110.4	33.4	55.5
**43.6**	0.14	2.5		821.1	209.1	12.0	219.2	28.4	23.7
**44.7**	0.10	3.3	3.55	715.8	192.6	11.6	233.6	27.6	21.7
**45.7**	0.11	2.4		984.8	318.8	14.0	206.1	25.4	22.6
**46.7**	0.10	1.4		810.6	316.3	10.2	119.3	27.7	42.6
**47.7**	0.11	1.5	3.5	592.3	160.3	7.4	88.2	28.4	59.0
**48.7**	0.09	2.3	3.47	679.5	156.0	8.1	68.0	30.6	82.7
**49.7**	0.10	2.4	3.57	680.6	256.2	8.0	111.2	27.8	45.8
**50.6**	0.09	2.6	3.41	748.3	236.8	8.6	90.7	32.6	65.8

Average depth (cm)	Bulk density (g cm^{-3})	Ash (%)	pH pore water	Ca (mg kg^{-1})	Mg (mg kg^{-1})	Sr (mg kg^{-1})	Ti (mg kg^{-1})	Pb (mg kg^{-1})	EF_{Pb}
**51.6**	0.09	2.5		712.3	197.0	8.5	75.7	31.6	76.6
**52.6**	0.08	2.6	3.55	617.2	252.9	7.9	92.6	25.1	49.7
**53.7**	0.10	3.9	3.51	642.5	233.6	8.5	122.5	35.0	52.3
**54.7**	0.11	4.6	3.52	398.7	104.8	5.7	189.0	16.2	15.7
**55.7**	0.07	2.8	3.49	313.9	111.9	4.7	128.3	13.2	18.8
**56.7**	0.07	2.4	3.74	572.8	104.6	7.3	149.0	28.6	35.2
**57.8**	0.07	2.3	3.52	567.7	99.8	7.3	133.5	26.7	36.6
**58.9**	0.07	4.0	3.73	642.1	99.6	8.2	175.3	37.1	38.9
**59.9**	0.06	2.2		506.7	110.5	6.7	171.7	23.0	24.5
**60.7**	0.06	2.1	3.44	559.3	88.9	7.9	187.8	30.3	29.6
**61.5**	0.07	4.8		617.3	101.5	8.1	209.1	26.3	23.1
**62.5**	0.07	4.0	3.54	516.7	94.7	8.1	264.8	25.3	17.5
**63.6**	0.07	3.1	3.6	489.9	75.8	8.5	284.2	22.6	14.6
**64.5**	0.08	3.9	3.53	410.5	71.0	6.1	171.9	29.5	31.4
**65.5**	0.08	2.4	3.55	390.9	99.8	5.7	157.9	24.1	28.0
**66.6**	0.09	7.1	3.51	352.8	61.6	6.2	221.8	29.7	24.5
**67.6**	0.06	4.5	3.54	410.4	101.5	8.4	372.2	25.4	12.5
**68.5**	0.09	5.3	3.66	511.1	114.0	8.3	265.2	36.4	25.2
**69.4**	0.11	7.2	3.79	368.4	60.5	7.4	290.7	36.1	22.8
**70.3**	0.11	8.2		339.5	80.1	10.3	473.5	29.1	11.3
**71.3**	0.11	7.7	3.64	406.8	99.2	10.3	540.8	32.9	11.2
**72.3**	0.13	6.1	3.55	268.8	83.7	7.9	388.6	25.5	12.0
**73.4**	0.11	5.7	3.69	233.6	94.7	6.4	306.3	23.3	14.0
**74.3**	0.13	4.9	3.52	331.6	123.1	9.0	453.8	35.2	14.2
**75.3**	0.12	5.9	3.51	235.1	53.3	6.6	366.6	27.9	13.9
**76.5**	0.14	6.0	3.62	230.0	80.0	7.8	399.1	24.7	11.4
**77.7**	0.13	4.0	3.53	214.9	75.8	6.7	342.9	19.8	10.6
**78.8**	0.12	6.6	3.61	217.4	55.5	5.3	242.6	12.5	9.4
**79.8**	0.12	6.0	3.85	240.8	109.9	8.0	399.9	12.5	5.7
**80.7**	0.12	6.4	3.59	168.0	55.4	6.7	410.0	9.9	4.4
**81.7**	0.13	5.8	3.44	216.1	82.9	6.7	351.0	12.1	6.3
**82.7**	0.15	6.6	3.54	202.0	82.4	6.0	324.7	9.6	5.4
**83.7**	0.14	6.2		203.4	57.1	7.0	428.8	14.2	6.1
**84.6**	0.13	5.8		199.3	177.6	10.6	717.7	16.4	4.2
**85.6**	0.11	4.4	3.58	218.4	129.3	9.8	588.8	12.4	3.9
**86.6**	0.12	3.9		142.7	177.5	4.2	183.8	11.3	11.3
**87.6**	0.13	3.6	3.53	175.9	152.9	7.8	409.3	17.7	7.9
**88.7**	0.11	3.9	3.57	161.3	152.0	7.0	340.6	16.1	8.7
**89.7**	0.13	4.6	3.5	173.6	159.6	7.7	411.8	16.8	7.5
**90.7**	0.11	2.9	3.54	202.2	176.5	11.3	637.5	17.5	5.0
**91.8**	0.13	7.0		256.1	202.0	12.4	645.4	17.1	4.8
**92.8**	0.15	7.7	3.71	184.1	156.3	9.1	493.1	16.1	6.0
**93.8**	0.13	5.6	3.62	208.3	148.6	10.5	633.4	13.4	3.9
**94.7**	0.13	6.1		161.1	165.8	7.7	430.2	15.9	6.8
**95.5**	0.13	7.0		185.5	151.9	9.8	692.4	11.7	3.1
**96.4**	0.11	5.3		160.8	180.8	5.8	283.7	9.4	6.1
**97.3**	0.12	2.1		141.8	132.7	6.5	395.8	9.7	4.5
**98.2**	0.12	3.6		115.9	281.4	4.7	217.7	6.8	5.8
**99.1**	0.13	6.9	3.54	174.3	113.7	10.2	679.5	8.4	2.3

Average depth (cm)	Bulk density (g cm^{-3})	Ash (%)	pH pore water	Ca (mg kg^{-1})	Mg (mg kg^{-1})	Sr (mg kg^{-1})	Ti (mg kg^{-1})	Pb (mg kg^{-1})	EF_{Pb}
**100.1**	0.11	3.9	3.63	151.3	173.9	7.2	429.4	6.2	2.6
**101.1**	0.11	3.6		160.2	121.6	8.9	586.7	6.8	2.1
**101.6**	0.12	2.9		168.4	157.7	8.1	459.0	3.6	1.4
**103.8**	0.11	3.4		174.3	197.5	7.4	377.8	3.2	1.6
**107.1**	0.11	4.2		190.5	213.2	8.8	471.3	3.7	1.4
**110.3**	0.13	5.5		239.6	364.6	15.2	942.4	3.7	0.7
**113.6**	0.11	4.0		156.1	224.7	6.7	333.3	2.9	1.6
**116.8**	0.10	2.5		240.4	258.0	9.4	506.3	2.7	1.0
**120.1**	0.09	4.2		271.8	171.5	11.4	502.7	3.4	1.2
**123.3**	0.10	1.5		198.8	76.5	7.1	316.0	2.8	1.6
**126.6**	0.13	3.8		163.9	135.9	6.7	321.2	2.2	1.3
**129.9**	0.13	2.7		213.0	74.5	7.7	311.3	3.0	1.8
**133.1**	0.11	1.7		257.0	139.6	7.1	231.7	2.8	2.2
**136.4**	0.10	0.8		170.9	53.5	4.1	143.3	2.0	2.5
**139.6**	0.11	1.3		165.0	85.2	5.8	241.8	2.2	1.7
**142.9**	0.11	1.3		163.6	78.4	3.8	147.5	2.3	2.8
**146.2**	0.10	1.0		232.9	86.1	5.3	140.7	3.4	4.5
**149.4**	0.10	1.4		173.4	99.2	3.6	121.4	2.5	3.7
**151.9**	0.10	0.5		178.3	108.0	5.1	165.8	3.1	3.5
**153.9**	0.09	1.5		229.7	68.2	5.3	175.2	3.7	3.8
**156.8**	0.12	3.0		184.0	94.1	5.0	234.5	3.0	2.4
**159.6**	0.10	3.3		211.5	64.2	4.7	220.9	2.8	2.3
**162.5**	0.12	2.7		248.7	69.1	6.3	193.5	4.2	4.0
**165.4**	0.11	1.8		246.0	124.4	4.6	162.4	4.0	4.5
**168.3**	0.12	2.3		286.1	155.9	7.5	259.1	5.0	3.5
**171.2**	0.15	1.7		257.2	103.1	4.7	119.4	3.2	5.0
**174.1**	0.11	1.8		222.1	88.5	3.1	79.3	2.4	5.6
**177.0**	0.09	1.2		293.1	90.1	5.7	83.0	2.1	4.6
**179.8**	0.10	2.0		335.2	126.1	7.7	129.8	1.5	2.2
**182.7**	0.11	2.3		241.9	132.9	4.6	128.2	1.4	2.1
**185.6**	0.09	0.7		367.2	220.0	4.6	45.5	1.8	7.3
**188.5**	0.08	1.7		430.3	169.1	5.4	49.0	1.3	4.9
**191.4**	0.09	6.4		342.3	180.7	7.7	631.0	1.8	0.5
**194.3**	0.09	2.6		491.7	251.5	6.5	70.4	0.8	2.0
**197.2**	0.07	2.0		642.4	244.1	7.6	33.3	0.7	3.8
**200.0**	0.08	2.3		786.3	460.9	9.6	52.6	0.8	2.9
**201.6**	0.08	2.3		849.4	508.0	11.1	169.0	1.4	1.5
**203.7**	0.08	1.7		838.2	338.0	9.7	59.3	0.7	2.1
**206.9**	0.06	1.9		1067.2	539.2	12.4	75.3	0.9	2.3
**210.1**	0.06	2.1		663.8	186.0	7.3	29.8	0.9	5.4
**213.3**	0.08	2.8		712.3	203.0	8.5	62.9	1.2	3.6
**216.5**	0.07	2.8		849.8	213.4	9.5	37.6	1.9	9.2
**219.7**	0.07	3.6		789.3	242.3	9.3	75.9	2.8	6.9
**222.9**	0.09	4.7		557.3	135.5	7.6	163.8	4.4	5.0
**226.1**	0.10	4.1		683.4	257.4	7.9	76.5	7.0	16.7
**229.3**	0.12	4.3		423.0	142.5	8.1	226.2	11.7	9.5
**235.6**	0.15	7.9		530.2	119.7	8.5	406.0	11.3	5.1
**238.8**	0.15	18.8		337.8	101.7	10.8	1150.5	19.3	3.1

Supplementary Table S3a – Pollen, NPP and spore types data (%).

**Average depth (cm)**	**3.6**	**5.5**	**7.6**	**9.6**	**11.8**	**13.7**	**15.6**	**17.8**	**22.9**	**26.0**	**30.1**	**32.0**	**33.5**	**35.4**	**38.5**	**40.5**	**43.6**	**45.7**	**48.7**	**50.6**	**53.7**	**55.7**	**58.9**	**60.7**	**63.6**	**65.5**	**68.5**
Abies	1.0	0.2	0.6	2.5	0.6	0.7	0.6	2.4	0.7	1.0	0.9	0.7	1.3	1.1	0.6	3.3	3.0	3.9	4.8	8.4	1.5	11.7	17.4	15.5	8.6	5.8	2.3
Fagus	2.4	5.2	2.3	2.2	1.1	1.7	0.6	2.0	0.7	1.0	1.0	2.2	5.3	10.1	13.5	9.2	13.3	10.4	16.1	11.1	24.9	8.8	2.2	4.8	8.2	10.8	14.4
Larix	4.0	4.7	7.4	11.5	2.1	2.7	2.9	5.7	3.9	1.5	2.0	2.1	2.4	0.9	1.0	2.3	2.9	3.3	5.0	3.5	5.1	2.1	1.4	1.1	7.3	5.0	6.6
Picea	43.2	46.2	56.3	21.7	80.4	71.2	75.5	60.9	66.6	87.3	49.9	37.1	29.2	27.9	25.3	41.9	47.2	63.7	38.8	43.3	14.3	64.0	75.5	66.3	51.3	51.4	36.6
Pinus cembra	1.6	0.5	0.4	1.8	0.2	4.4	1.2	2.4	5.1	0.4	1.9	0.9	10.4	5.3	1.8	1.3	2.0	2.0	6.0	2.3	2.3	2.1	1.1	0.7	1.9	0.9	1.7
Pinus	29.7	36.2	19.6	44.3	14.9	27.5	27.7	24.9	17.0	18.1	15.8	26.6	196.7	132.5	449.9	42.6	30.5	52.4	155.9	78.6	26.7	15.3	12.7	14.1	14.3	15.2	7.5
Ulmus	0.0	0.2	0.1	0.0	0.0	0.0	0.0	0.1	0.1	0.0	0.0	0.1	0.0	0.0	0.2	0.0	0.0	0.0	0.0	0.2	0.0	0.0	0.0	0.0	0.2	0.0	0.1
Alnus	5.0	3.7	3.7	7.9	2.5	2.3	2.7	3.8	2.3	0.6	7.7	10.8	7.3	8.5	13.5	7.5	6.0	1.4	7.6	5.3	9.0	1.9	0.1	1.0	3.8	4.0	4.3
Betula	0.4	0.3	0.1	1.6	0.6	0.6	0.8	0.4	0.7	0.0	1.6	0.8	1.8	1.1	2.4	3.3	3.0	0.2	1.0	0.0	2.5	0.2	0.1	0.1	0.3	1.3	0.9
Castanea	0.0	1.0	0.0	0.4	0.6	0.3	0.3	0.4	0.3	0.0	0.3	1.7	0.9	1.4	1.6	1.0	0.3	0.2	0.3	0.4	0.9	0.3	0.2	0.1	0.0	0.8	0.9
Corylus	2.4	3.9	1.4	3.6	0.8	1.4	1.5	0.4	0.8	0.3	0.7	0.7	0.4	1.8	1.6	1.3	2.1	1.4	2.0	3.2	1.9	0.6	0.2	0.2	1.1	0.5	0.4
Juglans	1.0	0.2	0.1	0.7	0.0	0.1	0.0	0.5	0.1	0.0	0.3	0.0	0.7	0.9	0.6	1.0	0.0	0.5	0.5	0.4	1.5	0.3	0.1	0.5	0.2	0.2	1.0
Juniperus-type	0.8	1.0	0.0	0.4	0.2	0.3	0.2	0.3	0.0	0.0	1.2	0.8	2.4	0.7	0.2	1.0	1.0	0.2	0.8	0.4	1.3	0.5	0.0	0.1	0.9	0.1	0.8
Olea europea	1.0	0.5	0.4	2.7	0.0	0.1	0.5	0.4	0.1	0.0	0.9	0.4	0.4	0.0	0.2	0.4	0.1	0.0	0.0	0.0	0.0	0.0	0.1	0.0	0.0	0.0	0.3
Populus	0.0	0.0	0.0	0.0	0.0	0.0	0.0	0.0	0.0	0.0	0.0	0.0	0.0	0.0	0.0	0.0	0.0	0.0	0.0	0.0	0.0	0.0	0.0	0.0	0.2	0.0	0.0
Salix	0.2	0.2	0.0	0.0	0.0	0.3	0.0	0.0	0.0	0.1	0.0	0.0	0.4	1.1	0.0	0.1	0.1	0.0	0.0	0.0	0.1	0.1	0.0	0.0	0.0	0.1	0.1
Sorbus-type	0.0	0.0	0.0	0.0	0.0	0.0	0.0	0.0	0.1	0.0	0.1	0.0	0.0	0.0	0.0	0.0	0.3	0.0	0.0	0.0	0.1	0.0	0.0	0.0	0.0	0.1	0.1
Vitis	0.0	0.0	0.0	0.0	0.0	0.0	0.0	0.0	0.0	0.0	0.0	0.0	0.4	0.2	0.0	0.0	0.0	0.2	0.0	0.4	0.3	0.0	0.0	0.0	0.2	0.2	0.1
Acer	0.0	0.0	0.3	0.0	0.0	0.0	0.0	0.0	0.0	0.0	0.1	0.0	0.0	0.2	0.0	0.0	0.0	0.0	0.0	0.0	0.0	0.0	0.0	0.0	0.0	0.0	0.0
Carpinus betulus	0.0	0.3	0.4	0.5	0.2	0.1	0.5	0.0	0.3	0.0	0.0	0.0	0.0	0.0	0.0	0.0	0.0	0.0	0.0	0.2	0.3	0.0	0.0	0.0	0.0	0.0	0.0
Fraxinus excelsior-type	0.2	0.5	0.1	0.7	0.2	0.1	0.0	0.0	0.0	0.0	0.0	0.0	0.0	0.0	0.0	0.0	0.0	0.0	0.0	0.0	0.0	0.0	0.0	0.0	0.0	0.0	0.0
Fraxinus ornus	2.2	3.1	1.7	1.1	0.6	1.0	0.8	1.5	1.6	0.5	0.7	0.8	0.9	1.1	1.4	0.8	0.5	0.6	0.0	0.9	1.3	0.3	0.0	0.2	0.6	1.0	0.5
Ostrya-type	8.8	9.0	11.6	23.3	2.5	5.2	3.2	3.2	1.9	0.3	0.6	1.4	1.8	2.5	1.0	3.3	1.6	0.6	1.5	2.8	3.0	1.1	0.0	0.7	0.8	1.7	1.4
Quercus robur-type	2.2	2.9	1.0	2.3	0.2	0.3	1.1	1.4	0.8	0.4	1.2	0.7	1.1	3.7	1.4	2.0	1.6	1.2	1.3	1.8	2.9	0.5	0.3	0.3	1.6	1.6	1.9
Tilia	0.0	0.0	0.1	0.0	0.0	0.0	0.3	0.0	0.0	0.0	0.0	0.0	0.0	0.0	0.0	0.0	0.1	0.0	0.0	0.0	0.1	0.1	0.0	0.0	0.0	0.0	0.0
Calluna vulgaris	71.9	29.2	24.6	37.3	98.7	14.0	24.8	11.2	16.2	8.8	30.8	3.2	1.3	0.2	0.4	0.1	0.4	0.0	0.3	0.2	0.1	0.0	0.0	0.2	0.5	0.1	0.1
Ericaceae	0.4	0.8	0.0	0.5	0.0	0.4	0.3	0.7	0.4	0.4	0.7	0.7	0.2	1.1	1.2	0.6	1.0	0.2	0.8	0.4	1.1	0.2	0.0	0.1	0.3	0.7	1.5
Cannabaceae	1.2	0.6	0.6	0.5	0.2	0.0	0.2	0.0	0.0	0.0	1.9	5.3	3.3	3.2	0.6	1.0	0.3	0.0	0.0	0.2	0.4	0.1	0.0	0.0	0.2	0.3	0.8
Centaurea cyanus	0.0	0.0	0.0	0.0	0.0	0.0	0.0	0.0	0.0	0.0	0.0	0.0	0.4	0.0	0.2	0.1	0.0	0.0	0.0	0.0	0.0	0.0	0.0	0.0	0.0	0.0	0.1
Cerealia	0.4	0.2	0.0	0.2	0.0	0.1	0.0	0.3	0.4	0.0	0.3	1.4	2.0	0.5	1.4	0.8	0.1	0.5	0.0	0.7	0.5	0.1	0.0	0.2	0.5	0.1	0.6
Plantago lanceolata-type	1.2	0.5	0.4	0.4	0.2	0.3	0.3	0.8	0.9	0.0	1.3	1.3	1.3	0.9	0.6	0.8	0.3	0.5	0.5	0.4	0.9	0.3	0.0	0.2	0.3	0.0	0.5
Plantago major-media-type	0.6	0.2	0.0	0.2	0.2	0.0	0.0	0.3	0.1	0.1	0.6	0.3	0.4	0.5	0.8	0.3	0.3	0.3	0.0	0.2	0.0	0.2	0.0	0.0	0.1	0.0	0.2
Rumex acetosa	0.0	0.0	0.0	0.2	0.0	0.1	0.0	0.0	0.1	0.0	0.0	0.0	0.0	0.0	0.0	0.0	0.0	0.0	0.0	0.0	0.0	0.0	0.0	0.0	0.0	0.0	0.1
Rumex acetosella	0.4	0.2	0.1	0.0	0.0	0.3	0.2	0.4	0.1	0.1	0.4	0.5	0.2	0.0	0.0	0.8	0.1	0.0	0.0	0.2	0.5	0.0	0.0	0.3	0.0	0.5	0.9
Secale	0.0	0.0	0.0	0.0	0.0	0.0	0.0	0.1	0.1	0.1	0.7	0.3	0.2	0.5	0.4	0.4	0.8	0.3	0.3	0.2	0.8	0.1	0.0	0.1	0.3	0.1	0.4
Urtica	3.0	2.6	0.9	2.0	0.2	0.3	0.5	0.3	0.1	0.0	1.3	0.7	1.8	3.7	0.2	0.8	0.3	0.2	0.0	0.4	0.4	0.1	0.0	0.3	0.2	0.7	1.5
Apiaceae	0.4	0.2	0.0	0.0	0.0	0.0	0.0	0.0	0.0	0.1	0.1	1.1	0.0	0.5	0.4	0.4	0.8	0.3	0.3	0.2	0.5	0.0	0.0	0.0	0.2	0.2	0.1
Asteraceae	0.8	0.3	0.1	0.5	0.0	0.0	0.0	0.4	0.5	0.0	0.7	0.4	1.1	0.0	0.4	0.3	0.4	0.0	0.3	0.2	0.1	0.0	0.0	0.2	0.5	0.2	0.2
Achillea-type	0.0	0.0	0.0	0.0	0.0	0.0	0.0	0.0	0.0	0.0	0.0	0.0	0.0	0.0	0.0	0.1	0.0	0.0	0.0	0.0	0.0	0.0	0.0	0.0	0.0	0.0	0.0
Brassicaceae	0.0	0.3	0.1	0.0	0.0	0.0	0.0	0.0	0.0	0.0	0.0	0.1	0.2	0.0	0.2	0.0	0.0	0.0	0.0	0.0	0.0	0.0	0.0	0.0	0.0	0.0	0.1
Caryophyllaceae	0.2	0.0	0.0	0.0	0.2	0.0	0.0	0.0	0.0	0.0	0.0	0.7	0.2	0.5	0.2	0.0	0.3	0.0	0.8	0.0	0.0	0.1	0.1	0.0	0.0	0.0	0.0
Cichoriaceae	0.2	0.0	0.1	0.0	0.0	0.1	0.0	0.0	0.3	0.0	0.1	1.2	0.2	0.5	0.2	0.0	0.1	0.5	1.0	1.4	0.3	0.0	0.0	0.0	0.5	0.1	0.2
Mentha-type	0.0	0.0	0.0	0.0	0.0	0.0	0.0	0.1	0.0	0.1	0.1	0.1	0.0	0.0	0.2	0.0	0.1	0.0	0.0	0.0	0.0	0.0	0.0	0.0	0.0	0.1	0.1
Poaceae	10.8	7.4	4.4	7.9	4.2	2.7	4.1	7.0	8.4	3.1	11.6	11.6	7.7	11.7	14.1	8.6	5.5	6.2	4.5	7.7	15.9	2.6	0.9	4.7	6.5	7.9	13.7
Potentilla-type	0.2	0.0	0.0	0.0	0.0	0.0	0.0	0.0	0.0	0.0	0.0	0.0	0.0	0.0	0.0	0.0	1.2	0.0	0.0	0.0	0.0	0.0	0.0	0.0	0.0	0.0	0.0

Average depth (cm)	3.6	5.5	7.6	9.6	11.8	13.7	15.6	17.8	22.9	26.0	30.1	32.0	33.5	35.4	38.5	40.5	43.6	45.7	48.7	50.6	53.7	55.7	58.9	60.7	63.6	65.5	68.5
Ranunculaceae	0.6	0.3	0.3	0.0	0.0	0.1	0.2	0.0	0.3	0.0	0.0	0.0	0.2	0.2	1.0	0.3	0.1	0.0	0.5	0.4	0.0	0.0	0.0	0.0	0.1	0.3	0.1
Ranunculus acris-type	0.0	0.2	0.0	0.0	0.0	0.0	0.2	0.1	0.0	0.0	0.1	0.3	0.2	0.2	0.2	0.0	0.0	0.0	0.3	0.0	0.0	0.0	0.0	0.0	0.0	0.0	0.1
Rosaceae	0.0	0.2	0.3	0.0	0.0	0.4	0.0	0.3	0.1	0.0	0.1	0.1	0.2	0.2	0.6	0.3	0.1	0.0	0.5	0.0	0.0	0.0	0.0	0.1	0.0	0.0	0.4
Rubiaceae	0.0	0.0	0.0	0.0	0.0	0.0	0.2	0.0	0.0	0.1	0.0	0.0	0.4	0.5	0.2	0.3	0.0	0.0	0.0	0.0	0.0	0.0	0.0	0.0	0.0	0.0	0.1
Saxifraga oppositifolia-type	0.0	0.0	0.0	0.0	0.0	0.0	0.0	0.0	0.1	0.0	0.0	0.1	0.0	0.0	0.0	0.1	0.0	0.0	0.0	0.0	0.0	0.0	0.0	0.0	0.0	0.0	0.0
Saxifraga stellaris-type	0.0	0.0	0.0	0.0	0.0	0.0	0.0	0.0	0.0	0.0	0.0	0.0	0.0	0.0	0.0	0.0	0.0	0.0	0.0	0.0	0.0	0.0	0.0	0.0	0.0	0.0	0.0
Saxigrafa granulata-type	0.0	0.0	0.0	0.0	0.0	0.0	0.2	0.0	0.0	0.0	0.0	0.0	0.0	0.0	0.0	0.0	0.0	0.0	0.0	0.0	0.0	0.0	0.0	0.0	0.0	0.0	0.0
Scrophulariaceae	0.0	0.0	0.0	0.0	0.0	0.0	0.2	0.0	0.0	0.0	0.0	0.0	0.2	0.0	0.2	0.0	0.3	0.0	0.5	0.4	0.4	0.1	0.0	0.7	0.0	0.5	0.5
Thalictrum	0.0	0.0	0.0	0.0	0.0	0.1	0.0	0.1	0.0	0.0	0.0	0.3	0.7	0.2	0.0	0.3	0.0	0.0	0.0	0.2	0.1	0.1	0.0	0.0	0.1	0.0	0.1
Cercophora	0.0	0.0	0.0	0.0	0.0	0.0	0.0	0.0	0.0	0.0	0.0	0.3	0.2	0.5	0.0	0.0	0.1	0.0	0.0	0.0	0.0	0.0	0.0	0.0	0.0	0.0	0.1
Podospora	0.0	0.0	0.0	0.0	0.0	0.0	0.0	0.0	0.0	0.0	0.0	0.3	0.7	0.0	0.0	0.0	0.1	0.0	0.0	0.0	0.0	0.0	0.0	0.0	0.0	0.0	0.0
Sordaria	0.0	0.0	0.0	0.0	0.0	0.0	0.0	0.0	0.0	0.0	0.1	1.3	0.4	2.3	0.0	0.3	0.0	0.0	0.0	0.0	0.0	0.1	0.0	0.0	0.0	0.0	0.0
Sporormiella	0.0	0.0	0.0	0.0	0.0	0.3	0.8	0.0	0.3	0.3	0.1	6.6	4.6	0.9	1.4	0.6	2.9	0.0	0.0	0.2	0.5	0.0	0.0	0.1	0.1	0.2	0.4
Botrychium	0.0	0.0	0.0	0.0	0.0	0.0	0.0	0.0	0.0	0.1	0.1	0.0	0.0	0.0	0.0	0.0	0.0	0.2	0.3	0.0	0.3	0.0	0.0	0.0	0.2	0.0	0.2
Dryopteris	0.0	0.5	0.4	0.4	0.2	0.6	0.5	1.5	0.7	0.8	0.4	1.6	0.7	3.2	2.6	0.7	0.3	0.0	2.8	1.4	2.0	0.8	0.2	0.8	0.9	0.8	1.3
Pteridium	0.0	0.0	0.0	0.0	0.0	0.0	0.0	0.0	0.0	0.1	0.1	0.3	0.2	0.7	0.4	1.8	0.7	0.5	0.3	0.4	0.1	0.8	0.4	0.6	1.1	0.9	0.9
Selaginella selaginoides	0.0	0.0	0.0	0.0	0.6	0.7	0.0	0.3	0.3	0.4	0.3	0.8	0.0	0.2	0.8	0.3	1.6	0.2	0.5	0.5	0.0	0.5	0.0	0.0	0.6	0.1	0.2
Sphagnum	0.8	1.3	0.6	0.4	0.6	0.6	1.1	0.1	0.0	0.0	0.6	0.3	0.0	0.0	3.8	2.0	0.5	0.0	0.0	0.0	0.3	0.0	0.0	0.0	0.0	0.0	0.0
Botryococcus	0.0	0.0	0.0	0.0	0.0	0.0	0.0	0.0	0.0	0.0	0.0	0.0	0.0	0.0	0.0	0.0	0.0	0.0	0.0	0.0	0.0	0.0	0.0	0.0	0.0	0.0	0.0
Cyperaceae	11.2	12.3	5.0	12.5	6.1	8.2	14.8	21.8	25.1	20.9	94.9	40.7	7.1	7.3	9.9	13.0	10.6	5.3	6.8	12.3	21.3	9.2	1.5	15.1	9.1	7.2	17.5

Supplementary Table S3b – Pollen, NPP and spore types data (%).

Average depth (cm)	70.3	73.4	75.3	78.8	80.7	83.7	85.6	88.7	90.7	92.8	98.2	102.7	107.1	112.5	117.9	124.4	135.3	145.1	152.9	163.5	173.1	183.7	192.3	201.6	212.2	222.9	233.5	239.9
Abies	3.3	1.3	2.4	4.6	3.2	3.2	4.8	8.0	10.4	6.7	6.7	12.4	8.7	4.3	10.2	6.5	7.0	1.9	1.4	2.8	1.4	1.5	1.3	0.1	0.0	0.0	0.1	0.2
Fagus	19.7	23.4	17.2	12.5	13.9	19.8	7.1	13.8	9.1	13.6	14.4	7.7	11.5	18.4	10.2	11.9	8.7	6.6	6.3	5.9	1.5	4.8	2.2	2.9	5.7	3.4	3.5	0.8
Larix	8.9	2.9	2.6	1.9	2.5	2.6	1.5	1.3	1.3	0.7	0.0	0.0	0.1	0.1	0.2	0.5	0.0	0.0	0.0	0.0	0.0	0.0	0.0	0.0	0.0	0.1	0.0	0.0
Picea	22.1	29.0	27.3	40.1	38.0	34.8	68.8	55.1	51.8	38.7	45.5	67.0	56.0	44.0	68.1	54.4	68.2	76.8	78.9	77.6	81.8	82.1	82.2	77.5	70.2	64.4	65.6	64.7
Pinus cembra	1.0	0.8	0.7	1.4	0.8	0.9	1.5	1.1	0.4	0.7	1.1	0.4	1.1	0.1	0.6	0.1	0.7	1.9	0.5	1.1	1.1	0.7	0.6	0.9	0.7	0.4	0.2	0.1
Pinus	8.7	5.0	5.9	9.6	7.6	6.4	9.5	6.4	12.1	7.6	5.7	10.5	5.5	7.3	7.5	10.0	9.0	11.2	8.3	11.7	10.7	12.6	7.3	8.0	6.6	6.3	6.8	13.7
Ulmus	0.1	0.1	0.0	0.4	0.0	0.3	0.0	0.0	0.0	0.1	0.5	0.0	0.1	0.2	0.3	0.3	0.3	0.1	0.3	0.7	0.3	0.1	0.1	0.3	0.6	1.2	1.0	1.2
Alnus	8.2	12.8	16.3	15.8	15.2	15.5	6.6	8.1	10.1	16.9	16.4	4.5	11.4	18.4	4.0	10.4	5.5	6.1	6.4	3.3	3.1	3.5	2.7	5.1	6.3	6.3	8.1	5.9
Betula	2.0	1.3	2.7	1.9	3.2	0.8	1.0	1.5	1.6	1.3	1.9	0.4	1.2	1.0	0.4	0.5	0.5	0.1	0.8	0.4	1.2	0.8	0.5	0.1	0.6	0.2	0.4	0.2
Castanea	2.2	0.7	0.5	2.0	1.2	0.2	0.2	0.1	0.0	0.0	0.0	0.0	0.0	0.0	0.0	0.0	0.0	0.0	0.0	0.0	0.0	0.0	0.0	0.0	0.0	0.0	0.0	0.0
Corylus	0.9	1.6	1.1	1.1	1.2	1.7	0.7	0.6	0.9	3.9	1.5	0.3	0.3	1.0	0.2	1.0	0.7	0.6	1.4	0.8	0.8	0.8	0.3	1.0	1.1	0.9	1.6	4.2
Juglans	0.4	0.2	0.2	0.2	0.4	0.5	0.3	0.6	0.0	0.2	0.0	0.0	0.0	0.0	0.0	0.0	0.0	0.0	0.0	0.0	0.0	0.0	0.0	0.0	0.0	0.0	0.0	0.0
Juniperus-type	0.8	0.6	0.5	0.3	0.8	0.0	0.0	0.6	0.4	0.0	0.6	0.0	0.2	0.0	0.0	0.0	0.0	0.0	0.0	0.0	0.0	0.0	0.0	0.0	0.0	0.0	0.0	0.0
Olea europea	0.0	0.0	0.2	0.1	0.0	0.1	0.2	0.0	0.0	0.0	0.0	0.0	0.0	0.0	0.0	0.0	0.0	0.0	0.1	0.0	0.0	0.0	0.0	0.0	0.0	0.0	0.0	0.0
Populus	0.0	0.0	0.0	0.0	0.0	0.0	0.0	0.0	0.0	0.0	0.0	0.0	0.0	0.0	0.0	0.0	0.0	0.0	0.0	0.0	0.0	0.0	0.0	0.0	0.0	0.0	0.0	0.0
Salix	0.0	0.0	0.1	0.2	0.0	0.0	0.0	0.0	0.0	0.0	0.0	0.1	0.0	0.0	0.0	0.0	0.0	0.0	0.0	0.0	0.0	0.0	0.1	0.0	0.0	0.0	0.0	0.2
Sorbus-type	0.3	0.0	0.1	0.1	0.1	0.0	0.0	0.0	0.1	0.0	0.0	0.0	0.1	0.2	0.0	0.0	0.1	0.1	0.2	0.3	0.0	0.0	0.0	0.1	0.0	0.0	0.0	0.0
Vitis	0.0	0.0	0.1	0.0	0.2	0.0	0.0	0.0	0.0	0.3	0.2	0.1	0.1	0.0	0.0	0.0	0.0	0.0	0.1	0.0	0.0	0.2	0.1	0.0	0.0	0.0	0.0	0.0
Acer	0.0	0.0	0.0	0.0	0.0	0.0	0.0	0.0	0.0	0.3	0.0	0.1	0.0	0.1	0.1	0.4	0.1	0.1	0.0	0.0	0.3	0.0	0.2	0.1	0.4	0.0	0.0	0.0
Carpinus betulus	0.0	0.1	0.5	0.0	0.6	0.5	0.1	0.0	0.0	0.6	0.4	0.1	0.2	0.4	0.2	0.5	0.4	0.1	0.0	0.1	0.0	0.0	0.0	0.0	0.0	0.0	0.0	0.0
Fraxinus excelsior-type	0.0	0.0	0.0	0.0	0.0	0.0	0.0	0.0	0.0	0.1	0.1	0.0	0.0	0.0	0.0	0.0	0.0	0.0	0.0	0.0	0.0	0.0	0.0	0.1	0.0	0.2	0.1	0.0
Fraxinus ornus	0.4	0.4	0.2	0.2	0.4	0.4	0.1	0.4	0.2	0.5	0.1	0.2	0.3	0.4	0.0	0.8	0.4	0.1	0.1	0.1	0.0	0.2	0.0	0.1	0.0	0.0	0.0	0.0

Average depth (cm)	70.3	73.4	75.3	78.8	80.7	83.7	85.6	88.7	90.7	92.8	98.2	102.7	107.1	112.5	117.9	124.4	135.3	145.1	152.9	163.5	173.1	183.7	192.3	201.6	212.2	222.9	233.5	239.9
Ostrya-type	2.1	2.7	2.5	1.2	1.9	3.5	0.7	1.4	1.2	1.4	0.7	0.6	1.5	0.4	0.4	0.8	0.2	0.3	0.3	0.2	0.4	0.3	0.2	0.0	0.4	0.2	0.8	0.0
Quercus robur-type	2.0	4.8	3.4	4.0	2.7	4.0	2.3	1.2	2.7	3.1	2.6	1.3	1.2	2.6	1.4	3.5	2.4	2.1	0.6	2.1	0.4	0.9	0.5	1.1	0.8	1.5	1.0	0.2
Tilia	0.0	0.1	0.0	0.0	0.0	0.0	0.0	0.0	0.1	0.0	0.0	0.0	0.1	0.0	0.0	0.2	0.0	0.0	0.1	0.1	0.2	0.2	0.4	0.1	0.3	0.3	0.3	1.1
Calluna vulgaris	0.3	0.2	0.2	0.1	0.1	0.0	0.4	0.1	0.0	0.0	0.0	0.0	0.0	0.0	0.0	0.0	0.0	0.0	0.0	0.0	0.0	0.0	0.0	0.0	0.0	0.0	0.0	0.0
Ericaceae	0.3	0.3	0.2	0.3	0.1	0.2	0.1	0.1	0.2	0.3	0.1	0.3	0.1	0.1	0.0	0.0	0.2	0.1	0.0	0.0	0.0	0.1	0.0	0.1	0.0	0.0	0.2	0.3
Cannabaceae	0.8	0.1	0.4	0.5	0.3	0.4	0.0	0.1	0.1	0.2	0.3	0.0	0.2	0.2	0.0	0.1	0.0	0.0	0.2	0.0	0.0	0.0	0.0	0.0	0.0	0.1	0.0	0.0
Centaurea cyanus	0.1	0.0	0.0	0.0	0.0	0.0	0.0	0.0	0.0	0.0	0.0	0.0	0.0	0.0	0.0	0.0	0.0	0.0	0.0	0.0	0.0	0.0	0.0	0.0	0.0	0.0	0.0	0.0
Cerealia	1.0	0.1	0.1	0.2	0.0	0.0	0.0	0.1	0.0	0.1	0.0	0.0	0.0	0.0	0.0	0.0	0.0	0.0	0.0	0.0	0.0	0.0	0.0	0.0	0.0	0.0	0.0	0.1
Plantago lanceolata-type	1.1	0.0	0.4	0.7	0.3	0.0	0.5	0.2	0.3	0.2	0.2	0.0	0.1	0.1	0.2	0.1	0.0	0.0	0.0	0.0	0.0	0.0	0.0	0.0	0.0	0.0	0.0	0.0
Plantago major-media-type	0.0	0.0	0.0	0.0	0.0	0.0	0.0	0.0	0.1	0.0	0.0	0.0	0.0	0.0	0.0	0.0	0.0	0.0	0.0	0.0	0.0	0.0	0.0	0.0	0.0	0.0	0.0	0.0
Rumex acetosa	0.0	0.0	0.0	0.0	0.0	0.0	0.0	0.0	0.0	0.0	0.0	0.0	0.1	0.0	0.0	0.0	0.0	0.0	0.0	0.0	0.0	0.0	0.0	0.0	0.0	0.0	0.0	0.0
Rumex acetosella	0.5	0.1	0.0	0.0	0.1	0.0	0.0	0.0	0.0	0.0	0.0	0.0	0.0	0.0	0.0	0.0	0.0	0.0	0.0	0.0	0.0	0.0	0.0	0.0	0.0	0.0	0.0	0.0
Secale	0.3	0.4	0.3	0.0	0.0	0.1	0.0	0.1	0.0	0.0	0.0	0.0	0.0	0.0	0.0	0.0	0.0	0.0	0.0	0.0	0.0	0.0	0.0	0.0	0.0	0.0	0.0	0.0
Urtica	0.9	0.0	1.8	0.1	1.1	0.0	0.2	0.2	0.1	0.0	0.2	0.2	0.1	0.1	0.1	0.1	0.0	0.0	0.0	0.0	0.0	0.0	0.0	0.0	0.0	0.0	0.0	0.0
Apiaceae	0.4	0.3	0.3	0.3	0.0	0.2	0.2	0.1	0.0	0.2	0.0	0.1	0.3	0.2	0.0	0.2	0.0	0.0	0.0	0.1	0.1	0.0	0.0	0.0	0.2	0.1	0.1	1.6
Asteraceae	0.3	0.3	0.2	0.3	0.1	0.2	0.1	0.1	0.2	0.3	0.1	0.3	0.1	0.1	0.0	0.0	0.2	0.1	0.0	0.0	0.0	0.1	0.0	0.1	0.0	0.0	0.2	0.3
Achillea-type	0.3	0.0	0.1	0.0	0.1	0.0	0.1	0.0	0.0	0.0	0.0	0.0	0.0	0.0	0.0	0.1	0.0	0.0	0.0	0.0	0.0	0.0	0.1	0.0	0.0	0.0	0.0	0.1
Brassicaceae	0.0	0.0	0.0	0.0	0.2	0.0	0.0	0.0	0.0	0.1	0.0	0.0	0.0	0.0	0.0	0.0	0.0	0.0	0.0	0.0	0.0	0.0	0.0	0.0	0.0	0.0	0.0	0.0
Caryophyllaceae	0.0	0.2	0.0	0.1	0.0	0.0	0.0	0.0	0.0	0.0	0.0	0.0	0.0	0.1	0.0	0.0	0.0	0.1	0.0	0.0	0.0	0.0	0.0	0.0	0.0	0.1	0.1	0.0
Cichoriaceae	0.7	0.2	0.1	0.3	0.3	0.0	0.1	0.0	0.2	0.1	0.1	0.0	0.1	0.1	0.3	0.0	0.2	0.1	0.1	0.0	0.1	0.0	0.0	0.1	0.1	0.1	0.3	0.1
Melampyrum	0.0	0.0	0.0	0.0	0.0	0.0	0.0	0.0	0.0	0.0	0.0	0.0	0.0	0.0	0.0	0.0	0.0	0.0	0.0	0.0	0.0	0.0	0.0	0.0	0.0	0.1	0.0	0.0
Mentha-type	0.0	0.0	0.1	0.1	0.0	0.0	0.0	0.0	0.0	0.1	0.0	0.0	0.0	0.1	0.0	0.0	0.0	0.0	0.0	0.1	0.1	0.0	0.0	0.0	0.0	0.0	0.0	0.0
Poaceae	12.1	9.0	10.7	4.9	6.0	5.6	1.5	3.6	6.2	4.3	3.2	0.9	2.5	2.8	1.5	2.3	1.7	1.7	1.2	3.6	6.1	3.3	3.6	7.2	10.1	15.2	4.3	3.8
Potentilla-type	0.0	0.0	0.0	0.0	0.0	0.0	0.0	0.0	0.0	0.0	0.0	0.0	0.0	0.0	0.0	0.0	0.1	0.0	0.1	0.0	0.0	0.0	3.4	0.0	0.0	0.0	0.0	0.0
Ranunculaceae	0.4	0.1	0.1	0.4	0.1	0.0	0.0	0.2	0.1	0.1	0.1	0.0	0.1	0.2	0.2	0.1	0.0	0.3	0.0	0.1	0.1	0.1	0.0	0.1	0.1	0.0	0.3	0.0
Ranunculus acris-type	0.0	0.0	0.2	0.1	0.0	0.0	0.0	0.0	0.0	0.0	0.0	0.0	0.0	0.0	0.1	0.0	0.0	0.0	0.0	0.0	0.0	0.0	0.0	0.0	0.0	0.0	0.0	0.1
Rosaceae	0.0	0.4	0.3	0.2	0.1	0.4	0.0	0.0	0.1	0.8	0.2	0.2	0.1	0.3	0.1	0.4	0.1	0.1	0.1	0.1	0.2	0.3	0.2	0.6	0.5	3.3	1.6	0.0
Rubiaceae	0.1	0.1	0.2	0.0	0.0	0.2	0.0	0.3	0.0	0.0	0.0	0.0	0.0	0.0	0.0	0.0	0.0	0.0	0.0	0.0	0.0	0.0	0.0	0.0	0.1	0.0	0.0	0.0
Saxifraga oppositifolia-type	0.1	0.1	0.1	0.0	0.0	0.0	0.0	0.0	0.0	0.0	0.0	0.1	0.0	0.0	0.0	0.0	0.0	0.0	0.0	0.0	0.0	0.0	0.0	0.0	0.0	0.0	0.0	0.0
Saxifraga stellaris-type	0.0	0.0	0.0	0.1	0.0	0.0	0.0	0.0	0.0	0.0	0.2	0.0	0.0	0.0	0.0	0.0	0.0	0.0	0.0	0.0	0.0	0.0	0.0	0.0	0.0	0.0	0.0	0.0
Saxigrafa granulata-type	0.0	0.0	0.2	0.0	0.0	0.0	0.0	0.0	0.0	0.0	0.2	0.0	0.1	0.0	0.0	0.0	0.0	0.0	0.0	0.0	0.0	0.0	0.0	0.0	0.0	0.0	0.1	0.0
Scrophulariaceae	1.2	0.0	0.1	0.2	0.0	0.0	0.0	0.0	0.0	0.1	0.0	0.0	0.0	0.0	0.0	0.0	0.0	0.0	0.1	0.0	0.0	0.0	0.0	0.0	0.0	0.0	0.0	0.1
Thalictrum	0.1	0.1	0.2	0.0	0.2	0.1	0.1	0.0	0.0	0.0	0.2	0.0	0.1	0.0	0.0	0.0	0.0	0.0	0.1	0.0	0.1	0.0	0.1	0.0	0.0	0.0	0.0	0.1
Cercophora	0.1	0.0	0.0	0.0	0.0	0.0	0.0	0.0	0.0	0.0	0.0	0.0	0.0	0.0	0.0	0.0	0.0	0.0	0.0	0.0	0.0	0.0	0.0	0.0	0.0	0.0	0.0	0.0
Podospora	0.0	0.0	0.0	0.0	0.0	0.0	0.0	0.0	0.0	0.0	0.0	0.0	0.1	0.0	0.0	0.0	0.0	0.0	0.0	0.0	0.0	0.0	0.0	0.0	0.0	0.0	0.0	0.0
Sordaria	0.0	0.0	0.0	0.0	0.0	0.0	0.0	0.0	0.0	0.0	0.0	0.0	0.0	0.0	0.0	0.0	0.0	0.0	0.0	0.0	0.0	0.0	0.0	0.0	0.0	0.0	0.0	0.0
Sporormiella	0.3	0.0	0.1	0.2	0.0	0.0	0.0	0.0	0.0	0.0	0.0	0.0	0.0	0.0	0.0	0.0	0.0	0.0	0.0	0.0	0.0	0.0	0.0	0.0	0.0	0.0	0.0	0.0
Botrychium	0.0	0.1	0.0	0.0	0.0	0.0	0.0	0.0	0.0	0.0	0.0	0.0	0.0	0.1	0.0	0.0	0.0	0.1	0.0	0.0	0.0	0.0	0.0	0.3	0.0	0.1	0.0	0.2
Dryopteris	2.3	1.7	2.9	0.8	1.9	0.6	1.1	0.3	1.2	0.8	0.8	0.1	1.3	1.7	0.4	1.2	0.5	2.2	1.6	0.7	0.8	0.7	0.6	1.1	1.4	1.2	2.6	8.9
Pteridium	1.1	0.8	0.7	0.4	0.5	0.0	0.5	0.5	0.7	0.3	0.4	0.1	0.1	0.3	0.3	0.2	0.1	0.1	0.0	0.0	0.1	0.2	0.1	0.5	0.2	0.0	0.0	0.0
Selaginella selaginoides	0.1	0.1	0.0	0.0	0.0	0.0	0.1	0.3	0.0	0.1	0.0	0.2	0.0	0.2	0.0	0.0	0.0	0.1	0.1	0.0	0.0	0.0	0.0	0.0	0.0	0.0	0.0	0.0
Sphagnum	0.1	0.2	0.0	0.0	0.1	0.0	0.0	0.0	0.2	0.1	0.0	0.0	0.0	0.1	0.0	0.0	0.0	0.0	0.0	0.0	0.0	0.0	0.0	2.1	9.4	85.9	3.9	5.8
Botryococcus	0.0	0.0	0.2	0.5	1.1	0.0	0.1	0.0	1.1	47.6	20.0	0.0	4.6	0.0	7.0	112.3	28.6	0.0	0.2	0.1	0.0	0.4	0.0	0.0	0.0	0.0	0.0	0.0
Cyperaceae	29.5	18.4	31.9	12.1	14.7	12.9	8.2	24.5	13.1	14.6	7.1	11.4	4.4	5.7	2.8	18.7	4.5	2.2	0.5	3.6	8.8	7.0	7.9	26.7	39.9	30.3	10.5	2.5

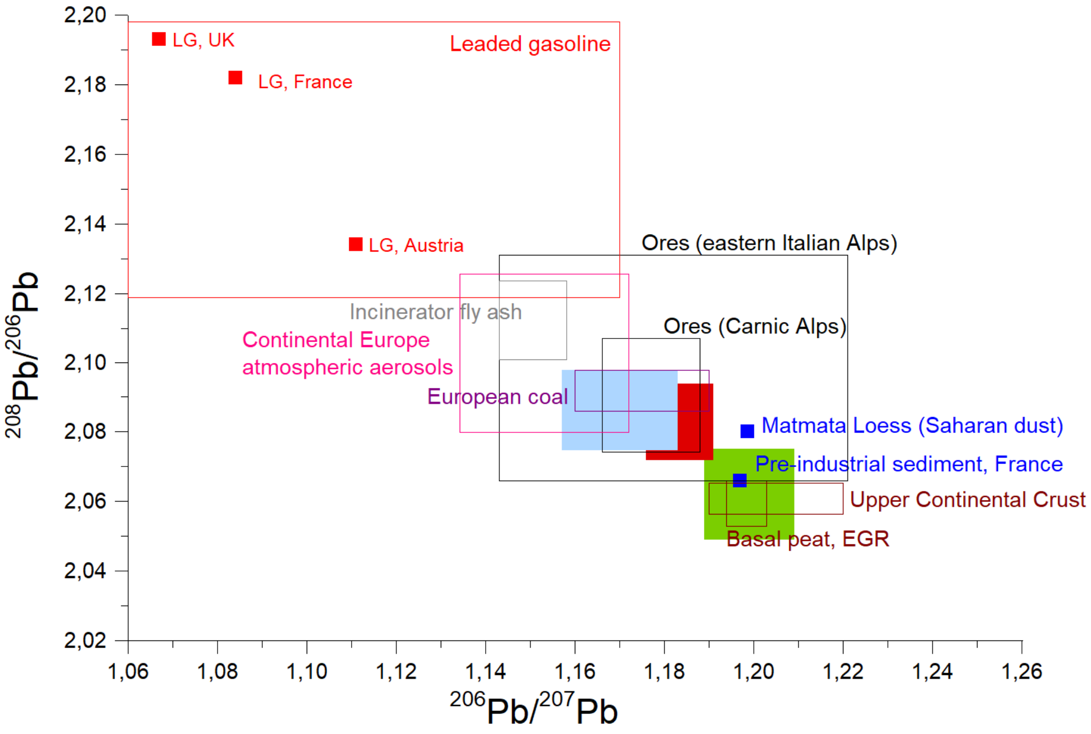

Supplementary Figure S2 – Three-isotope plot of $^{206}Pb/^{207}Pb$ *vs.* $^{208}Pb/^{206}Pb$ of this study (full coloured rectangles) with other studies (empty rectangles and points). The green, red and light blue rectangles represent a pre-contamination period, an early contamination period and the industrialized time, respectively. The isotopic values of the basal peat of Étang de la Gruère (EGR) in the Jura Mountains of Switzerland (Shotyk et al. 2001) and of pre-industrial sediment in France (Monna et al. 1997) are reported for comparison. As possible natural sources, the loess from the Sahara Desert (Grousset et al. 1994) and the Upper Continental Crust (Kramers and Tolstikhin 1997) are visible in the graph. Mining deposit isotopic values of the Carnic Alps (Artioli et al. 2016) and eastern Italian Alps (Nimis et al. 2012) are drawn, as a comparison with the early contamination period. For the industrialized period, study of coal combustion (Kylander et al. 2005; Komárek et al. 2008), atmospheric aerosols in the continental Europe (Monna et al. 1997; Flament et al. 2002), waste incinerator ash (Monna et al. 1997; Hansmann and Köppel 2000; Carignan et al. 2005; Kylander et al. 2005) and leaded gasoline (Hopper and Ross 1991; Leistel et al. 1997; Monna et al. 1997; Monna et al. 1999; Hansmann and Köppel 2000; Novák et al. 2003) are considered. See Table S4 for details.

Supplementary Table S4 – Pb isotope signatures of natural and anthropogenic Pb sources in Europe.

**Source**	**Site**	**$^{208}Pb/^{206}Pb$**	**$^{206}Pb/^{207}Pb$**	**References**
Matmata Loess	Southern Tunisia	2.0801	1.1988	(Grousset et al. 1994)
Upper continental crust (UCC)		2.0653-2.0564	1.19-1.22	(Kramers and Tolstikhin 1997)
Pre-industrial sediments	France	2.066	1.197	(Monna et al 1997)
EGR Basal peat (305-405 cm)	Switzerland	2.053-2.066	1.194-1.203	(Shotyk et al. 2001)
Ores	Eastern Italian Alps	2.066-2.131	1.143-1.221	(Nimis et al. 2012)
Ores	Carnic Alps	2.074-2.107	1.166-1.188	(Artioli et al. 2016)
European coal		2.086-2.098	1.16-1.19	(Kylander et al. 2005; Komárek et al. 2008)
Atmospheric aerosols	Continental Europe	2.08-2.1256	1.1342-1.1719	(Flament et al. 2002)
Incinerator fly ash	Continental Europe	2.1070-2.1236	1.143-1.1547	(Monna et al. 1997; Hansmann and Köppel 2000; Carignan et al. 2005; Kylander et al. 2005)
Leaded gasoline		2.1188-2.1980	1.06-1.17	(Leistel et al. 1997; Monna et al. 1999)
Leaded gasoline	Austria	2.134	1.111	(Hopper and Ross 1991)
Leaded gasoline	France	2.182	1.084	(Monna et al. 1997)
Leaded gasoline	UK	2.193	1.067	(Monna et al. 1997)

References

Brigo L, Camana G, Rodeghiero F, Potenza R (2001) Carbonate-hosted siliceous crust type mineralization of Carnic Alps (Italy-Austria). Ore Geol Rev 17:199–214

Brigo L, Dulski P, Möller P, Schneider H-J, Wolter R (1988) Strata-bound mineralizations in the Carnic Alps/Italy, in: Mineral Deposits within the European Community. Springer Berlin Heidelberg, pp 485–498

Carignan J, Libourel G, Cloquet C, Le Forestier L (2005) Lead isotopic composition of fly ash and flue gas residues from municipal solid waste combustors in France: implications for atmospheric lead source tracing. Environ Sci Technol 39:2018–2024

Casati P, Jadoul F, Nicora A, Marinelli M, Fantini Sestini N, Fois E (1982) Geologia della Valle dell'Ansiei e dei gruppi M. Popera-Tre Cime di Lavaredo (Dolomiti Orientali). Riv Ital di Paleontol e Stratigr 87:371–510 (in Italian)

Dal Cin R (1972) I conglomerati tardo-paleozoici post-ercinici delle Dolomiti. Mitteilungen der Gesellschaft der Geol und Bergbaustudenten Österreich 20:47–74 (in Italian)

Flament P, Bertho M-L, Deboudt K, Véron A, Puskaric E (2002) European isotopic signatures for lead in atmospheric aerosols: a source apportionment based upon 206 Pb/ 207 Pb ratios". Sci Total Environ 296:35–57

Grousset FE, Quetel CR, Thomas B, Buat-Menard P, Donard OFX, Bucher A (1994) Transient Pb isotopic signatures in the western European atmosphere". Environ Sci Technol 28:1605–1608

Hansmann W, Köppel V (2000) Lead-isotopes as tracers of pollutants in soils". Chem Geol 171:123–144

Hopper JF, Ross HB (1991) Regional source discrimination of atmospheric aerosols in Europe using the isotopic composition of lead. Tellus 43:45–60

Kral F (1988) Zur Wald-und Siedlungsgeschichte Osttirols: Pollenanalyse der Moore am Kartitscher Sattel. Bot. Jahrbücher für Syst Pflanzengeschichte und Pflanzengeographie 75:61–67

Kramers JD, Tolstikhin IN (1997) Two terrestrial lead isotope paradoxes, forward transport modelling, core formation and the history of the continental crust. Chem Geol 139:75-110

Leistel JM, Marcoux E, Thiéblemont D, Quesada C, Sánchez A, Almodóvar GR, Pascual E, Sáez R (1997) The volcanic-hosted massive sulphide deposits of the Iberian Pyrite Belt Review and preface to the Thematic Issue. Mineralium Deposita 33:2–30

Monna F, Lancelot J, Croudace IW, Cundy AB, Lewis JT (1997) Lead isotopic composition of airborne material from France and the Southern UK implications for Pb pollution sources in urban areas. Environ Sci Technol 31:2277–2286

Monna F, Dominik J, Loizeau J-L, Pardos M, Arpagaus P (1999) Origin and evolution of Pb in sediments of Lake Geneva (Switzerland-France). Establishing a stable Pb record". Environ Sci Technol 33:2850–2857

Novák M, Emmanuel S, Vile MA, Erel Y, Véron A, Pačes T, Wieder RK, Vaneček M, Štepánová M, Brízová E, et al. (2003). Origin of lead in eight Central European peat bogs determined from isotope ratios, strengths, and operation times of regional pollution sources. Environ Sci Technol 37:437–445
